# Cross-Neutralizing Antibodies in HIV-1 Individuals Infected by Subtypes B, F1, C or the B/Bbr Variant in Relation to the Genetics and Biochemical Characteristics of the *env* Gene

**DOI:** 10.1371/journal.pone.0167690

**Published:** 2016-12-09

**Authors:** Dalziza Victalina de Almeida, Karine Venegas Macieira, Beatriz Gilda Jegerhorn Grinsztejn, Valdiléa Gonçalves Veloso dos Santos, Monick Lindenmeyer Guimarães

**Affiliations:** 1 Laboratory of AIDS and Molecular Immunology, Oswaldo Cruz Institute - FIOCRUZ, Rio de Janeiro, Brazil; 2 National Institute of Infectology/ FIOCRUZ, Rio de Janeiro, Brazil; China Academy of Chinese Medical Sciences, CHINA

## Abstract

Various HIV-1 *env* genetic and biochemical features impact the elicitation of cross-reactive neutralizing antibodies in natural infections. Thus, we aimed to investigate cross-neutralizing antibodies in individuals infected with HIV-1 *env* subtypes B, F1, C or the B/Bbr variant as well as *env* characteristics. Therefore, plasma samples from Brazilian chronically HIV-1 infected individuals were submitted to the TZM-bl neutralization assay. We also analyzed putative N-glycosylation sites (PNGLs) and the size of gp120 variable domains in the context of HIV-1 subtypes prevalent in Brazil. We observed a greater breadth and potency of the anti-Env neutralizing response in individuals infected with the F1 or B HIV-1 subtypes compared with the C subtype and the variant B/Bbr. We observed greater V1 B/Bbr and smaller V4 F1 than those of other subtypes (p<0.005), however neither was there a correlation verified between the variable region length and neutralization potency, nor between PNLG and HIV-1 subtypes. The enrichment of W at top of V3 loop in weak neutralizing response viruses and the P in viruses with higher neutralization susceptibility was statistically significant (p = 0.013). Some other signatures sites were associated to HIV-1 subtype-specific F1 and B/Bbr samples might influence in the distinct neutralizing response. These results indicate that a single amino acid substitution may lead to a distinct conformational exposure or load in the association domain of the trimer of gp120 and interfere with the induction power of the neutralizing response, which affects the sensitivity of the neutralizing antibody and has significant implications for vaccine design.

## Introduction

A vaccine that aims to elicit strong HIV neutralizing antibodies (nAb) must overcome their genetic variability at least at the antigenic level. The neutralizing activity induced by HIV-1 should aid in the understanding of the immune response elicited by vaccine candidates [1–3]. Several studies have reported that antibodies from plasma obtained during chronic HIV-1 infection could potently neutralize primary isolates of HIV-1 and were able to neutralize genetically diverse and distinct HIV-1 strains [4–8]. These nAb primarily recognize five different epitopes on Env, including the CD4 biding site (CD4bs), V1/V2 loop, V3 loop, interface gp120/gp41 and the membrane-proximal external region (MPER) on gp41 [9–12].

In response to the constant HIV-1 genetic evolution, the epitope specificity of the nAb that is gradually developed during infection also influences the breadth of the nAb responses [13,14]. Some viral features, such as variable loop lengths and the number of glycosylation motifs, are associated with the neutralization breadth [3,15–17]. Therefore, the characterization of neutralization specificities for distinct subtypes is a difficult but critical process to accumulate knowledge and develop a successful vaccine.

In Brazil, HIV-1 subtypes B, their B/Bbr variants, F1 and C, as well as diverse recombinants evolving these subtypes are prevalent [18,19]. The B/Bbr variant, which represents 37 to 57% of HIV-1 subtype B strains in the country, differs from the pandemic subtype B by the substitution of the amino acid proline by a tryptophan at the top of the V3 loop of gp120 (GWGR instead of the classical GPGR) [18,20–22] and its antigenic characteristics [20,23,24]. HIV-1 subtype C is the most prevalent worldwide and is involved in 20 to 80% of HIV-1 infections in Southern Brazil [25]. This subtype is spreading in other Brazilian geographic regions, and most of these sequences formed a monophyletic cluster [26]. The F1 subtype has a prevalence of 8.4 to 24.4% in the Southeastern region of Brazil [27]. The F1 subtype is also highly prevalent in Romania [28] and Galicia [29] despite its reduced prevalence worldwide. In this context, the present study aimed to investigate possible *env* genetic characteristics related to broad and potent neutralization in plasma from individuals infected with HIV-1 predominant subtypes in Brazil.

## Materials and Methods

### Study group

HIV-1-infected patients undergoing clinical follow-up at the Evandro Chagas Nacional Institute of Infectious Diseases from the Oswaldo Cruz Foundation (INI-FIOCRUZ) were invited to participate in this study and selected for enrollment. The main criteria for inclusion were: having at least 6 months of HIV-1 infection, and plasma samples representing the following HIV-1 Brazilian subtypes (B, B/Bbr, F1 and C), which have been previously classified in other studies from our group, based on C2-V3 *env* region subtyping. All protocols in the present study were performed in accordance with institutional guidelines and resolutions and were approved by the Oswaldo Cruz Institute Ethics Committee (CAAE: 01080112.4.0000.5248). However, we were not able to obtain informed consent for all participants included in this study, but plasma samples have been de-identified prior to analysis in order to maintain participant confidentiality. Moreover, a confidentiality letter was signed by the research team responsible for the experiments, thus ensuring the patients anonymity.

### Full-length *env* Sequencing

The *env* gene was amplified from PBMC by touchdown PCR [30] under the following conditions: 94°C×2’ for one cycle; 94°C×30”, 64°C×45” (decreasing 0.2°C per cycle) and 68°C×2’ for 20 cycles; 94°C×30”, 60°C×45”, 68°Cx2’ for 20 cycles and a final extension cycle of 68°Cx10’. The outer primers were BC1s (AGAAATGGAGCCAGTAGATC)/*env*M, and the inner primers were *env*Atopo and *env*M [31]. Sequences were generated using the BigDye Terminator v.3.1 Cycle Sequencing Ready Reaction Kit with an automated ABI 3100 Genetic Analyzer (Applied Biosystems, CA, USA).

### Sequence analysis

Sequences were assembled and edited using the SeqMan software from the package DNASTAR Lasergene (MA, USA). Nucleotide and deduced amino acid sequences were initially aligned using ClustalW on Mega 6 [32] and then re-aligned with HXB2 on Gene Cutter tools of the HIV sequence database from Los Alamos National Laboratory (LANL). HIV-1 subtyping was obtained via the REGA HIV-1 subtyping tool [33] and confirmed using neighbor-joining phylogenetic trees from the *env* region. We also used the programs Variable Region Characteristics, N-linked glycosylation sites (PNLG) [34], and CATNAP (Compile, Analyze and Tally NAb Panels) [35]. For the analysis of HIV-1-specific signatures, VESPA (viral epidemiology signature pattern analysis) was used. All programs were available from LANL. For the analysis of HIV-1 subtype-specific and neutralization potency signatures, thresholds of 1.0 and 0.6 were used, respectively.

### Sequence data

The 51 HIV-1 sequences obtained in the present study are available in the GenBank database (accession numbers KX181891-KX181941‏).

### Pseudovirus (psV)

The psVB (plasmid Rhpa42597) [16] and psVC (plasmid Cap210.08) [15] from the NIH neutralization panel were selected based on minor genetic *env* distances to the Brazilian HIV-1 subtype B and C consensus 0.24 and 0.19 of divergence, respectively. Two pseudoviruses (psVGWGR and psVF1) were produced based on the consensus sequence obtained from Dambe software (http://dambe.bio.uottawa.ca/dambe.asp) using HIV-1 Env B/Bbr (n = 15) and F1 (n = 11) sequences. The psVGPGR was produced by site-directed mutagenesis of the tryptophan from the B/Bbr consensus sequence to the proline on the top of the V3 loop of gp120. All three consensus pseudovirus sequences were synthesized by GenScript ^™^ (NJ, USA), and amplicons were cloned into the expression vector pcDNA3.1DV5-His TOPO TA (Thermo-Fisher Scientific, MA, USA). The plasmids were expanded in *E*. *coli* Top10, extracted using Wizard Plus Miniprep DNA Purification Systems (Promega, WI, USA) and quantified in a Nanodrop (Wilmington, USA) spectrophotometer. The viral stocks of single round HIV-1 *env* psVs infection were produced by co-transfecting 293T/17 cells (ATCC, VA, USA) (70% of confluent cells in T75) with 4 μg of an HIV-1 rev/env expression plasmid and 10 μg of pSG3ΔEnv. For transfections, 50 μL P3000 reagent and 35 μL Lipofectamine 3000 (Lipofectamine^®^ 3000 reagent, Thermo-Fischer Scientific, MA, USA) were used in 715 μL of Opti-MEM^®^ Reduce Serum Medium for each mix. After optimization, we followed proceedings according to the manufacturer’s recommendation. Using *env* amplification, the psVs were sequenced to confirm that they exactly matched the initial sequences.

### Neutralization Assay

The 50% tissue culture infectious dose (TCID50) for each pseudovirus preparation was determined by infection of TZM-bl cells as previously described [16]. To determine the capacity of the assay to discriminate between neutralizing antibodies and possible plasma artifacts, we used normal human plasma samples and the plasmid murine leukemia virus (MuLV) *env* as controls. Plasma was inactivated after the neutralization assay at 56°C x 60’. TZM-bl cells were expanded and stored following the instructions provided at http://www.hiv.lanl.gov/content/nab-reference-strains/html/home.htm. TZM-bl is a HeLa cell that was engineered to express CD4 and CCR5 [36] and contains integrated reporter genes for firefly luciferase and *Escherichia coli* β-galactosidase under the control of an HIV-1 LTR [37], permitting sensitive and accurate measurements of the HIV-1 infection. The psVs (200 TCID50) were incubated with plasma in triplicate and added to TZM-bl cells in the presence of DEAE-dextran 20 μg/mL. Neutralizing antibodies titers were expressed by the reciprocal of plasma dilutions. The 50% inhibitory concentration (IC_50_) of the monoclonal antibodies (mAbs) 2F5, 2G12, 447-D, and CH01 and soluble CD4 inhibitor were used at a given range of dilutions (final concentration: 10 μg/mL), this experiment was repeated three times to generate the mean value. These values were measured and analyzed with Excel-based Macro [38]. The mAbs and sCD4 were obtained from the AIDS Research and Reference Program, Division of AIDS (DAIDS), National Institute of Allergy and Infectious Diseases (NIAID), National Institutes of Health (NIH).

### Statistics

The statistical analysis was performed using GraphPad Prism (V-5.01-GraphPad, USA). One-away ANOVA followed by Bonferroni's multiple comparisons test for correction were used to analyze significant differences between the means of geometric media sensibility (GMS) according to the psV and the nAb titers of the different plasma samples grouped by subtype and neutralization potency. Additionally, one-way ANOVA followed by Dunnett’s multiple comparison tests was used to evaluate the differences in the length of variable regions and the PNLG of gp160. To indicate that there might be significant associations between amino acids in particular positions in the alignment and the neutralization susceptibility of a given Env, contingency tables and respective statistics were used (i.e. chi-square or Fisher’s exact test for categorical variables). P-values less than 0.05 were considered statistically significant.

## Results

### HIV-1 *env* diversity and phylogeny

**Fig 1:**
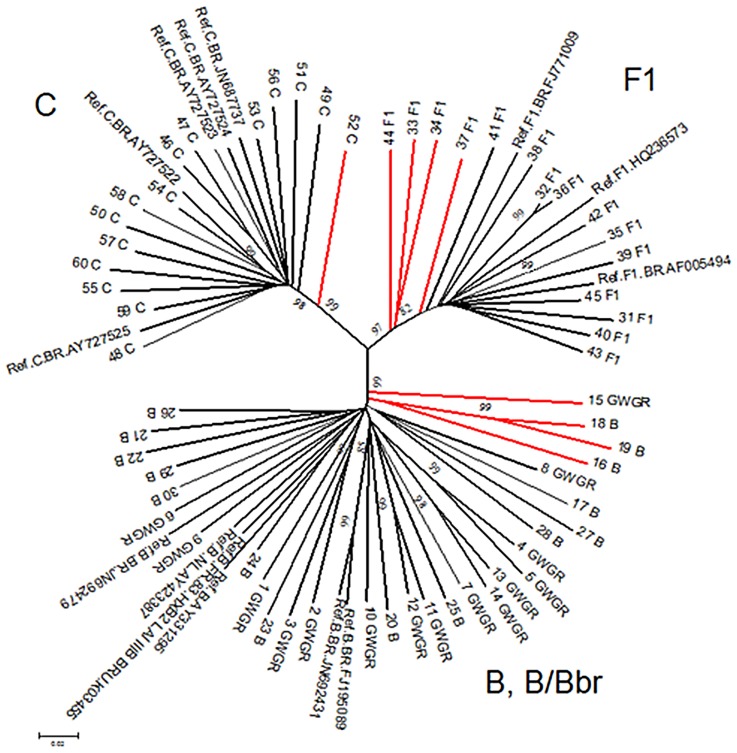
Phylogenetic tree of *env* gene (nt-2574) was generated by the Neighbor-Joining method using HIV-1 reference sequences (Ref). The bootstrap analysis was performed with 1000 replicates. The branches in red represent the recombinant samples.

Based on the REGA HIV subtyping tool and in the phylogenetic analyses of the 60 full-length *env* sequences (2.5 kb) from HIV-1 participants, 26 were reclassified as HIV-1 subtype B (12 HIV-1 B pandemic and 14 HIV-1 B/Bbr), 14 C subtype and 11 subtype F1 (Fig 1). In addition, nine subtypes, which were classified as HIV-1 unique recombinant forms (8 BF1 and 1 BC) were excluded from subsequent analysis. The following genetic divergence intersubypes/variants were observed: B and B/Bbr was 0.19, B-F1 = 0.31, B-C = 0.33, F1-C = 0.32, F1-B/Bbr = 0.32 and C-B/Bbr = 0.34.

### Neutralization phenotype

To characterize the neutralization phenotypes of HIV-1 Env-pseudoviruses (psVGWGR, psVGPGR and psVF1) obtained from Brazilian consensus sequences and those selected from the neutralization panel psVB (Rhpa) and psVC (Cap210), we characterized their phenotypes using mAbs and sCD4. The Brazilian psVs were inhibited by all mAbs and presented reduced IC_50_ geometric means when compared to psVB and psVC (Table 1). sCD4 neutralized all of the studied psVs, and mAbs 2F5, and CH01 were able to neutralize almost all of the psVs with the exception of the psVC and psVB, respectively. The mAbs 2G12 and 447-D inhibited only Brazilian psVs. Furthermore, the psVF1 had the strongest neutralization sensitivity for all mAbs.

**Table 1:** Mean inhibitory concentration (IC) 50 values (μg/mL) for triplicate assays with pseudoviruses (psVs) and the geometric mean (GM) as indicated.

	Mean IC_50_ in TZM-bl (μg/mL)				
	2F5	2G12	447_D	CH01	sCD4
**psVF1**	**0.10**	**0.06**	**0.01**	**0.05**	**1.09**
**psVGPGR**	**3.50**	**6.70**	**2.15**	**8.70**	**1.24**
**psVGWGR**	**4.00**	**3.05**	**8.72**	**3.30**	**0.15**
**psVB(Rhpa)**	**9.90**	**>10**	**>10**	**>10**	**3.70**
**psVC(Cap210)**	**>10**	**>10**	**>10**	**3.20**	**1.90**
**GM of detected**	**1.92**	**1.07**	**0.57**	**1.46**	**1.04**
**GM of all**	**4.24**	**6.57**	**4.51**	**3.40**	**1.04**
**% detected**	**80%**	**60%**	**60%**	**80%**	**100%**

Red ≤0.625 (1st Quartile), orange ≤3.000 (2nd), yellow ≤3.750 (3rd), green >3.750, white Undetected.

The results were plotted on CATNAP (http://hiv.lanl.gov/catnap). The cell color indicates the following categories: white, no neutralization (IC_50_>10 μg/mL); green, weak neutralization; orange and yellow, moderate neutralization; and red, strong neutralization. The psV MuLV was tested together as a negative control, and the IC_50_ was undetected.

### Association of breadth of neutralizing antibody potency to HIV-1 ENV subtypes

**Fig 2:**
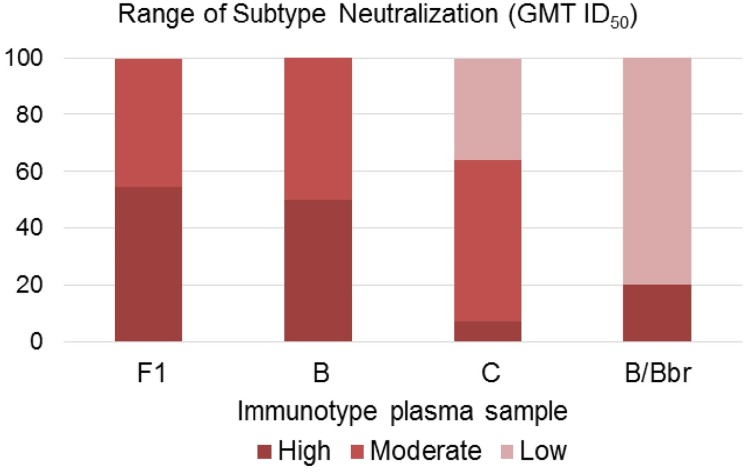
Neutralization range according to HIV-1 subtypes. The bars represent the percentage of potential neutralizing antibody for each plasma group, according to HIV-1 subtype.

The potential of the HIV-1 plasma samples to neutralize the psVs was displayed in magnitude sorting and grouped according to the geometric mean titer (GMT) ID_50_ values. Samples were grouped as with low neutralization potential (GMT 20–99), moderate potential (GMT 100–999) or high neutralization potential (GMT>1000) (S1 Table). Taken together, almost all of our 51 subjects exhibited nAb response; however, 18 (35%) of them had no nAb response for one or two psVs. Of the 51 plasma samples analyzed, 16 (31.4%) were classified as low potential, 19 (37.2%) as moderate potential and 16 (31.4%) as high neutralization potential (S1 Table). According to this analysis, plasma samples from all studied individuals infected with HIV-1 subtypes B and F1 presented high or moderate neutralization potency ID_50_ values, and most individuals from the B/Bbr variant and subtype C exhibited low and moderate neutralization potency, respectively (Fig 2). Additionally, the GMT of variant B/Bbr (144) was 11-fold smaller (p<0.001) than the GTM of subtype B samples (1605). This result reveals distinct immunogenic properties between HIV-1 subtypes. No significant nAb activity was observed when plasmas were tested against a negative control (psV MuLV).

### Cross neutralize reactive response to *env* psV

The psVGWGR had the strongest reactive response between analyzed psVs, with a geometric mean of sensibility (GMS) of 886, indicating similar sensitivity as psV tier 1. In addition, only one nAb B/Bbr plasma sample did not neutralize this psV. Interestingly, the GMS of psVGWGR is 3.7–fold higher compared with psVGPGR (p<0.01), even though they differ in only one single amino acid (W to P) (S1 Table). Following the sequence of susceptibility, the psVC (tier 2) exhibited a GMS of 572, which is approximately double the susceptibility of psVF1 (258), psVGPGR (238) and psVB (185) (S1 Table).

In addition to the potent nAb detected in subtype B (GMT 1605) and F1 plasma samples (GMT 777), broad cross neutralization to the psV was noted. Although subtype C plasma samples exhibited low neutralization antibody titers, the antibodies were more specific for the V3 GWGR epitope, and the GMT values from psVGWGR and psVC were 771 and 238, respectively (Table 2).

**Table 2:** Geometric mean titer of nAb from HIV-1 plasma samples B, B/Bbr, C and F1 against psVs.

	Plasma Samples			
	GMT B	GMT F	GMT C	GMT B/Bbr
psVGWGR	2833	1025	771	335
psVC	1577	1338	238	296
psVB	1977	631	25	68
psVF1	914	772	136	70
psVGPGR	1320	424	63	132
GMT	1605	777	180	144

Data from the neutralization assay of all psV evidenced by GMT of subtype plasma samples. (psV: pseudovirus; GMT: geometric mean titer).

**Fig 3:**
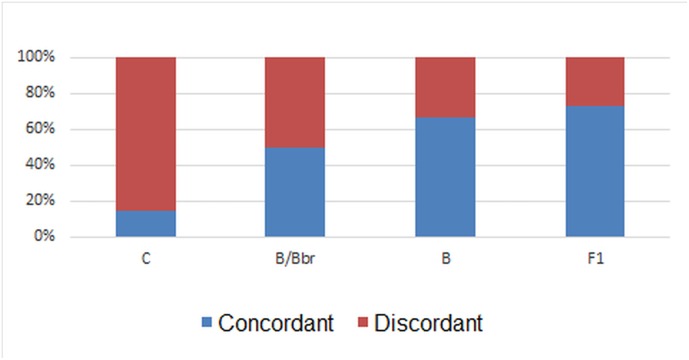
Dissonance of neutralization range between psVGWGR and psVGPGR. The bars represent the percentage of dissonance in neutralization range for each HIV-1 subtype.

To assess the impact of the tryptophan to proline substitution, we compared the disagreement in the neutralization ranges between psVGWGR and psVGPGR in each plasma subtype group. From this analysis, we note that the disagreement in neutralization ranges were as follows: 28% of discordance in plasma samples from subtype F1, 34% for B, 50% for B/Bbr and 86% for C (Fig 3).

### Analysis of the PNLG sites and variable regions of HIV-1 in plasma samples

**Fig 4:**
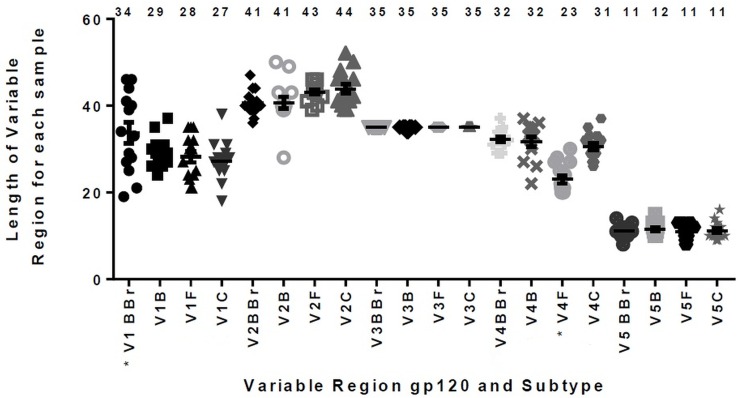
Comparison of variable region lengths among Brazilian HIV-1 (B, B/Bbr, F1 and C) subtypes. One-way ANOVA p-values in subsequent Dunnett’s multiple comparison tests indicating statistically significant difference (p<0.005) are marked with an asterisk. The horizontal bars at the top of each column indicate the means of length for each variable region.

Here, we analyzed the Env protein characteristics described to influence HIV-1 neutralization sensitivity, such as the number of PNLG and length of gp120 variable regions in HIV-1 plasma samples grouped by subtypes. Our results showed that the size of each gp120 variable region among HIV-1 subtypes had a statistically significant difference. We observed greater V1 B/Bbr and smaller V4 F1 than those of other subtypes (p < 0.005) (Fig 4). However, neither was a correlation verified between variable region length and neutralization magnitude, nor between PNLG and HIV-1 subtypes (Table 3).

**Table 3:** Number of potential N-linked glycosylation (PNLG) sites of HIV-1 Env sequences according to the range of neutralization potency and HIV-1 subtypes.

Group	Number of PNLG					
	Gp120		Gp41		Gp160	
	Min-Max	Mean	Min-Max	Mean	Min-Max	Mean
**High nAb**	**21–30**	**25.5**	**3–6**	**4.5**	**25–36**	**30.5**
**Low nAb**	**22–30**	**26.0**	**4–5**	**4.0**	**27–35**	**31.0**
**F1**	**21–31**	**26.0**	**4–7**	**5.5**	**25–38**	**31.5**
**B**	**22–30**	**26.0**	**3–5**	**4.0**	**26–35**	**30.5**
**B/Bbr**	**23–30**	**26.5**	**3–5**	**4.0**	**27–35**	**31.0**
**C**	**20–29**	**24.5**	**4–5**	**4.5**	**24–32**	**28.0**

### Neutralization signature patterns

Some authors have proposed that some Env features that elicit strong antibodies in natural infection might be useful to integrate vaccine design immunogens [39,40]. Thus, we verified possible association of some Env signatures patterns with neutralization potency and HIV-1 subtypes in an alignment of 937 amino acids sites containing all 51 ENV sequences (S2 Fig).

In order to verify the potential signatures related to neutralization susceptibility 16 Env sequences that presented a high neutralization range were compared with 16 sequences with lower neutralization ranges. From this analysis, three signatures were suggested to be enrichment, (68.8%) L14W (56.2%), (81.2%) P360W (68.8%), and (56.2%) R843H (62.5%). Here, the first amino acid represents high neutralization and the second amino acid represents low neutralization potency. The results of signature analyses were combined on contingency table (Chi-square or Fisher's exact tests) and only one statistically significant signature was identified, the site P360W (p = 0.013), position 313 in relation to HXB2.

**Fig 5:**
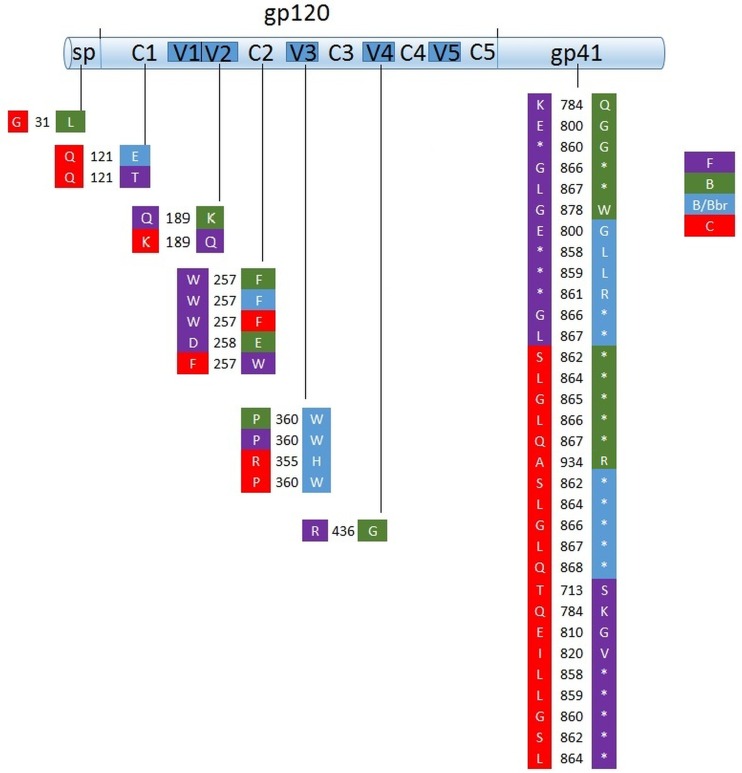
Scheme of viral envelope gene (gp120 and gp41) and representation of HIV-1 subtype-specific signatures. The asterisk indicates insertions or deletions. The numbers represent the position of each HIV-1 subtype-specific signatures in reference to amino acid alignment.

Concerning HIV-1 subtype signatures, we verified seven to twelve signatures (substitution or insertions) in a pair-to-pair comparison. These signatures were localized in the signal peptide, C1, V2, C2, V3, and V4, with the majority located in gp41 (Fig 5 and S1 Fig). From this analysis, comparing HIV-1 subtype F1 samples (GMT 777), which showed better neutralizing response than B/Bbr samples (GMT 144), we verified subtype-specific signatures located in regions C2, V3 and gp41, which might influence in the distinct neutralizing response.

## Discussion

We evaluated the neutralization breadth and potency of plasma samples from HIV-1-infected Brazilian individuals using a representative panel of psVs and attempted to correlate the antibody response to the genetic and biochemical characteristics of HIV-1 subtypes. Comparing the neutralizing phenotype of psVs, we verified that psVF1 and psVGPGR were the most cross-susceptible to the inhibitors. The resistance of psVC and psVB to some mAbs were also verified in others studies [39,41–44], and in the present study this resistance could be associated with escape mutations. In the mAb 2F5, which recognizes the ELDKWA epitope [45] from gp41, we verified a change from an alanine to a glutamine in psVC. For the 2G12 mAb, which recognizes the mannose residues N295, N332, N339, and N392 and the V4 loop in relation to HXB2 positions [46], we observed that some asparagine residues are absent in psVC and psVB, leading to a phenotypical resistant profile (S2 Fig, alignment residues: N321, N359, N366, and N419) [41,47–51]. Given that mAb 447-D is specific for viruses that carry the GPGR motif at the top of V3 loop [52], a strong neutralization of psVGPGR, psVB (Rhpa) and psV F1 was expected. However, the psVB (Rhpa) was not inhibited at 10 μg/mL (IC50), but in previous studies in concentrations of 48.9 μg/mL [52] and 46.2 μg/mL [53] a susceptibility was observed, indicating that this relevant epitope on the V3 loop was not easily exposed in this psV. The CH01 is a broad nAb used to target the second Env site of vulnerability by covering amino acid residues in V1V2 loops and sugar moieties at N160 (S2 Fig, N173 on V2) [53]. Given that this asparagine was present in all psVs, we suggested that the distinct neutralization susceptibility verified to CH01 could be associated with the V1V2 length [49–51]. All psVs were sensitive to sCD4, which causes irreversible shedding of gp120 from and subsequently inactivates Env [54]. Therefore, our results are in full agreement with previous studies, confirming the reliability and accuracy of the assay. We also emphasize that the use of broadly neutralization panel including psVs based on local HIV consensus sequences is of paramount importance to better characterize HIV humoral immune response.

Screening the neutralizing activity of a panel of 51 HIV-1 plasmas samples against the five psV, we observed nAbs in 31.4% of the analyzed samples, which is consistent with the 10 to 30% values detected in recent studies [55,56]. Herein, nAbs were detected in most of the samples from HIV-1 subtypes F1 and B (GPGR). Although these subtypes are genetically distant, they are correlated immunologically as verified by V3 peptide seroreactivity [57] and IFN-γ ELISpot response to Gag and Nef [58].

We have not determined whether the serum neutralization breadth observed here is specifically prevalent in the plasma samples using assays. However, by dissonance analysis, we observed that subtype C plasma samples exhibited a specific response to GWGR that is increased when compared with GPGR or GPGQ motifs present in V3 of the psVs. Thus, we suggest that the conformational change deriving from amino acid substitution (GWGR) could result in a better accessibility of the epitope. As previously described, the influence of the modified variable regions on the adjacent protomers results in altered access to nAbs [12,59,60]. Additionally, in general, broad serum neutralization is characterized by the presence of one or very few antibody specificities [10,45].

We detected an increased amount of N-glycosylation sites in plasma sample sequences of low neutralization range and psVs with minor GMS, however it was not statistically significant. This finding suggests a masking of the nAb epitope by glycans on the surface of Env, forming a ‘‘glycan shield” that reduces access to protein epitopes and nAb induction. According to van Gils et al., [60] an increase in the length of the V1V2 loop and the number of PNLG on the glycoprotein is directly associated with the protection of HIV-1 against HIV-specific neutralizing antibodies. In relation to the psVs, we observed that psVGWGR and psVGPGR had the same number of PNLG but discordant GMS to plasma samples, and we assume that nAb in the plasma samples were more directional to the top of the V3 loop.

Of the 937 amino acids compared between the 51 sequences, only three (L14W, P360W and R843H) amino acids positions were more frequent in a particular neutralizing response groups. The amino acid position 14, which is part of the signal peptide, plays a role in the efficiency of the protein secretion, in the orientation of Env protein to the membrane, impacting folding and the exit from the endoplasmic reticulum [61]. The amino acid change of proline to tryptophan at 360 position can directly interfere with the formation of bridging sheet and adjacent surfaces from the outer domain of gp120, and this also impact to V3-loop antibodies that block the binding of gp120–CD4 complexes [62]. Therefore, we observed that virus with W360 were more sensitive to neutralization and induced weak anti-Env response. The other signature pattern was observed on the cytoplasmic tail (R843H). The substitutions in this region can lead to effects on the binding of antibodies to the V1-V2 region, the V3 loop, or the C5 domain of gp120 [63]. This might suggest that alterations of amino acids composition in these regions (signal peptide, V3 loop and cytoplasmic tail) are an important determining factor in the induction of nAb, at least in our study population. Such changes could be influencing the expression or binding to antibodies in exposed regions of each protomer.

Currently, little is known about antibody affinity maturation in relation to the presented antigen. In this process, antibody-antigen interactions are of great importance for the selection of B cell characteristics, such as structural peptide size and charge of the amino acids surrounding the electrostatic forces (hydrogen bridges, hydrophobic interactions and Van der Waals force) [64]. Recently, Doria-Rose and Gordon report about the possibility of the recruitment of specific viral sequences to activate a "correct" BCR and facilitate the development of particular powerful antibodies [65].

The limitations of our study are the same shared by most authors working with neutralizing antibodies. In fact, studies addressing nAb could be influenced by host genetic characteristics, disease progression profile, HIV-1 viral load, and studies with small sample size due to the high costs of the experiments, especially in resource-limited settings. In our analysis, we considered only chronic HIV-1 infected individuals and explored viral characteristics such as HIV-1 subtypes, length of the variable regions, and differences on N-linked glycosylation sites (PNLG) that have been described to be implicated in the potency and breadth of nAb. We were able to observe that some individuals especially infected with HIV-1 subtypes B and F1 produce high titers of broadly reactive neutralizing antibodies, which are of particular interest for vaccine design. The presence of tryptophan instead of proline on the top of the V3 loop facilitates the exposure of the trimeric structural domain, contributing to viral neutralization. Therefore, it is important to highlight that these kinds of studies are able to increase understanding and add to the growing body of evidence that the antigenic and immunogenic properties of Env should facilitate the development of an effective HIV-1 vaccine.

## Supporting Information

S1 Fig Alignment of 51 HIV-1 envelope amino acid sequences according to B, B/Bbr F1 and C subtypes. (PDF)

S2 Fig Alignment of *env*-psVs in relation to the HXB2 reference virus. (PDF)

S1 Table Plasma samples from individuals infected with the different HIV-1 subtypes exhibit antibodies activity profiles against distinct pseudoviruses. (DOCX)
