# Supplementary material for: Cross-Neutralizing Antibodies in HIV-1 Individuals Infected by Subtypes B, F1, C or the B/Bbr Variant in Relation to the Genetics and Biochemical Characteristics of the env Gene
Source: PLoS One. 2016 Dec 9;11(12):e0167690. doi: 10.1371/journal.pone.0167690 (PMC5147934; doi:10.1371/journal.pone.0167690)
Supplement: S1 Fig — (PDF) [file pone.0167690.s001.pdf]

```
                    10         20         30         40         50         60         70         80         90         00
                                                                                                                              100
17B        MRAKGIRK-N WQVL-W---- --RWGTM--- LLGMLMICKA -A----DDSW VTVYYGVPVW KEATTTLFCA SDAKAYDTEA HNVWATHACV PTDPNPQEVV
20B        ..V.....-. C.R.-.---- --.G.I.LTM .........S. EE----.QW. .......... ...N...... .....H.... .......... ..........
21B        .....M..-H ..HW-.---- --TG.IL--- .........ST ------EQL. .......... R......... ......K..V .......... ........IP
22B        ..VR....-. C.H.-.---- --......--- .........S. -T----NQL. .......... R......... .........V .......... ......R..I
23B        ........-. ..H.-.KKGI MLQ..IL--- .........N. EE----EKT. ....F..... ...K...... ....GHS... .......... ....D....E
24B        ....E...-. CLL.-.---- --.G.IL--- .........S. -T----EKL. .......... R.T....... ......E..M .......... ..........
25B        ..VMET..-. Y.H.-.---- --...I.--- .........S. -.---EEQ.. .......... .......... ......E... ..I....... .........A
26B        ...R....-. Y.L.-.---- --...A.--- .........S. -T----EQL. .......... .......... .........V .......... .........E
27B        ..V..TG.-. Y.L.-.---- --...M.--- ....W...S. -T---KEE.. ..I....... .......... .......P.. ..I....... .........F
28B        ..V..MQR-. ..L.-G---- --K..L.LII .P.I.I..N. ------EKL. .......... .......... .......A.. .......... ....S....G
29B        ..V.....-S ..HS-.RWGT MLL....--- .........R. -T----EKL. .......... .......... .........G .......... ....K...I.
30B        .K......-. Y.P.-.---- --.G..V--- .........S. -T-----NL. .......... .......... ....T..P.G .......... ........I.
1B/Bbr     ..V...K.-. ..L.-G---- --G..I.--- .........S. -T----.NL. .......... ...N...... .......... .......... ........I.
2B/Bbr     ....E...-. C.L.-.---- --K.....--- ..K.....S. -T----EKL. .....K.... .......... .........V .......... ........IP
3B/Bbr     ....E...-. C.L.-.---- --KM....--- ..R.....S. -M----.QL. .....K.... .......... .........V .......... ..........
4B/Bbr     ...R.T..-. C.HW-.---- -WK..I.--- .........N. -R----.K.. .......... .......... .......... .......... .........K
5B/Bbr     ..VR...R-. Y.H.-.---- --...I.--- .........N. -T----EK.. .......... .......... .......... .......... ........GS
6B/Bbr     .K.R....-. ..HW-.---- -WK..I.--- .......YS. -E----.KL. .......... .......... ......T... ..I....... ..........
7B/Bbr     ....ET..-. C.HW-S---- --I...L--- FF.......S. EE----EKL. ...H...... .......... ......K... .......... ......R..P
8B/Bbr     ........-. C.L.-.---- --......--- .........S. -T----KEL. .......... .......... .......... .......... .........E
9B/Bbr     ...R.T..-. C.HS-.---- -W...IT--- .........S. -K----E.L. .......... ...S...... ...S.VK... ..I....... ....H...I.
10B/Bbr    ..V.E-KTGD ..HW-.---- -W......--- .........S. EE----.KL. .......... .......... .........V .......... ..........
11B/Bbr    ...TE.KR-. C.HSC.---- --......--- .........S. KE----.EF. .......... ..T....... .......... ..I....... ....D....E
12B/Bbr    ....E..R-. C.HSC.---- --...I.--- .........S. -V---REQL. .......... ..T....... .......... ..I....... .........E
13B/Bbr    ....E..R-. C.HW-.---- -WK..M.--- .........S. EE----.KL. .......... .......... .......... .......... .........K
14B/Bbr    ....E-IR-D C.HW-.---- -WK..M.--- .........S. EE----EKL. .......... ..T....... .......... .......... .........K
31F1       ..VR.MQR-. ..H.-G---- --K..LL--- F..I.I..N. -.-----.NL. .......... .......... ......EK.V ..I....... .........N
32F1       ..VR.MQR-. ..H.-G---- --K.SLL--- F..I.I..N. -T----GNL. .......... .......... .......K.V .......... .........W
35F1       ..VR.MQR-. ..H.-G---- --K..IF--- F..T.I..N. -T----GNL. .......... ...N...... ....S..N.. .......... ....D...L.
36F1       ..VR.MQR-. ..H.-G---- --K.SLL--- F..I.I..N. -T----GNL. .......... .......... .......K.V .......... .........W
38F1       ..VR.MQR-. .LH.-E---- --K..LL--- F..I.I..S. -.-----.NL. .......... ...K...... .......R.. .......... ........ML
39F1       ..VR.MQR-. CLHW-G---- --...LL--- F..I.I..N. ------ENL. .P........ .D........ ......EK.V .......... ........MD
40F1       ..VR.MQR-. Y.H.-G---- --K..LL--- F..I.I..N. -T----EK.. .......... .......... .........V .....S.... .........N
41F1       ..VM....-. C.H.-.RWGT MW.....--- .........R. -.---TEPL. .......... .......... .........V .......... ..........
42F1       ..VR.MQR-. ..N.-G---- --K..LF--- F..I.I..N. ------EKL. .......... ...S...... ....S....V .......... .........D
43F1       ..VR.MWR-. ..H.-G---- --K..LL--- F..I.I..N. -T----ENL. .......... ...N...... ......EK.V .......... .........S
45F1       ..VM....-. ..HW-G---- --K..LL--- F..I....N. -T----EKL. .......... R......... .......P.V .......... ......R.I.
46C        ..V...QR-. .KQW-.---- --I..IL--- GFWV..M.NV -T----GNL. .......... ...K...... .......Q.V ..I....... ........L.
47C        ..VM..QR-. .KQW-.---- --I..IL--- GFWV...YNV -T----GNL. .......... R..K...... .......Q.V ..I....... ....S...LM
48C        ..V...QR-. .KQW-.---- --I..IL--- GFWL....NV -M----GNL. .......... RD.K...... .........V .......... ....S.R.M.
49C        ..VR..QR-. .KQW-.---- --I..IL--- G.L.....NG -M----GNL. .......... .D.K...... .........V .......... ........M.
50C        ..VT..QR-. .RHW-.---- --I..IL--- GFW.V.V.NV -M----GNL. .......... .D.K...... L........V .......... ....D...LF
51C        ..VMET.R-. .KPW-.---- --I.SIL--- GFWL...YSV -K----GNL. .......... ...K...... ....G.E..V .......... ....S...LF
53C        ..V..TQR-. .KQW-.---- --I..VL--- GFWL....NG -K----ENL. .......... R..K...... ......E..V .......... ........MF
54C        ..V...QR-. .KQW-.---- --I..IL--- GFC--...NV -V----GNL. .......... ...K...... .........V .......... ........MD
55C        ..V..TQR-. .KQW-.---- --I..IL--- GFW...VYNV -M----GNL. .......... ...K...... ......E..V .......... ........M.
56C        ..VR..QR-. .RQW-.---- --I.SIL--- GFWL..V.NG -V----GNR. .......... ...K...... ........P.V .......... ........M.
57C        ..V...QR-. CKQW-.---- --I.IIL--- GFW....YNV -R----GNL. .......... ...K...... ......E..V .......... ........M.
58C        .....TQR-. YKQW-.---- --M..VL--- GFW.F..YNV -RGNEKENL. .......... ...K...... .........V ..I....... ........M.
59C        .KV...QR-. .KQW-.---- --I.VIL--- GFW...VYNV -R----GNL. .......... .D.N...... .........V .......... ........M.
60C        ..VR..QR-S .KQW-.---- --I..IL--- GFW...LYNV -R----GNL. .......... .D.KA..... .........V .......... ......H...

                     Sinal peptide                                      gp120
```

```
                  10         20         30         40         50         60         70         80         90         00
                                                                                                                          200
17B      LKNVTENFNM WKNNMVDQMQ EDVISLWDQS LKPCVKLTPF CVPLNCTDAT NNSTNSTN-- ---------- -----NSTNS TMDEEGGMKN CSFNV-TSIG
20B      .-........ ......E..H .......... .........I ..T....NVN TT.N..STG- ---------- ----NYNNSW EGV...EW.. ....ITA.L.
21B      .E....P... ...D..E..H ..I..I.... .........L ..T.H...V. .GNGTNG--- ---------- -------R.V KL.T..EI.. .....T...R
22B      .V........ .T....E..H Q.I.....E. .........L ..T...SSPI F.NS.NAS-- ---------- ------INGN WVNMTTE... ....IT...R
23B      .V........ .D.D...... ..I....... .........L ..V.S..NV. VTE..R.E-- ---------- -----TNGTI WENMK.EI.. .....TEKVK
24B      .E........ ......E... ..I....... .........L ..T...SN.N FTNA.A.K-- ---------- -----.NDST INEMNKE... ....IT.G..
25B      .E........ ......E..H ..I....... .........L ..T....NVK ..TSTN---- ---------- --------SN FSKM..DI.. ....ITS..E
26B      .D........ .........H ..I....... .........L ..T.K...VN .T.NSTNS-- ---------- ------NSSK .IE.V.EI.. .....T...R
27B      .......... .........H ..I....... .........L ..T...S.VK GTN.ANNTTA M--------- --GNSTN..G SLIG.TD... .....TKT..
28B      FE........ ......E..H T.I....... .........L ..T.....VK T.A.TTA--- ---------- ------.PSN SSW....... ....ITR.MK
29B      .E....E... ......E.LH ..I....... .........L ..T....NNI TITKDNSTSA NS-------- -TSLT...SL DNSTS.EI.. .....T..RS
30B      .E........ .....A...H ..I....... .........L ..T.E..NYE K..SSVN--- ---------- -------SS. WENMKEEI.. .....TN..R
1B/Bbr   .......... ......E..H ..I.N..... .........L ..T.....NN SSN------- ---------- ---------- NI.MKEE... ....IT.G..
2B/Bbr   .E........ ....I.E..H ..T....... .........L ..T.....LK .GTS..---- ---------- -------SSD EERMKEE... ....IT...K
3B/Bbr   .E........ ...D..E..H ..I....... .........L ..T.K..NWK KQNGT.ATGG ANT------- TSGTNTTN.D WGKM..E... W....T....
4B/Bbr   .......... ......E..H ..I....... .........L ..T....NYN .TNA...TTR GSNSTT--NS NITNSTKND. WI.MNEE... ....IT.G..
5B/Bbr   IE....K... ......E..H ..I....... .........L ..T....NV. MPLNCTNKNR TAKS-----N MTCNGTRQ.I .GTIGEE... ....AP.G.E
6B/Bbr   .G........ ......E..H ..I....... .........L ..T.....VV TCNST.ASNN SSTG------ TCNNTTPY.I SKEM..EI.. ....ITSQ.K
7B/Bbr   .E........ ......E..H ..I....... .........L ..I...SNLN .STNITS--- ---------- ------.DQG NL.MREEI.. ....IT.G..
8B/Bbr   ......D... ......E..H ..I....... .........L ..T...SNCN ITAD.T..-- ---------- ------TNAN SSC..M.... .....T.G..
9B/Bbr   .E....R... ......E..H ..I.....EG .........L ..T....P.D VTYATV.YDN ---------- ---VTYTNSM LQNITEE... ....IS.N.R
10B/Bbr  .E........ ......E..H .......... .........L ..T.E.ENV. K.TS------ ---------- ---------G EIQVKEEL.. .....T.G..
11B/Bbr  .V........ ......E..H ..I....... .........L ..I....NYN ATK..T.S-- ---------- -----.NSSE WENMR.EIQ. ....IT.G.E
12B/Bbr  .G..I..... ......E..H ..I....... .........L ..I.....YN ATK..A..TN T--------- ---TS.NSSD LDQMRREIQ. ....IP.G.E
13B/Bbr  .N........ ......E..H ..I....... .........L ..T.H..NYN .TNA...TTR GSNSTTNSNI TNSTKT.ND. WI.MNEE... ....IT.G..
14B/Bbr  .N........ ......E..H ..I....... .........L ..T.H..NYN .TNA...TTR GSNS------ ------TNAN SSC..M.... .....T.G..
31F1     .V......D. ......E..H T.I....... .........L ..T...S.SN TTTNDN.TTN ---------- ----A.RNST LWE.P.AIQ. ...HLT.QVR
32F1     .E........ .N....E..H T.I....... .........L ..T.H..NFN A.G.QNSTL- ---------- -----TQNST LKE.PDAIQ. ....MT.ELR
35F1     .E........ ...D..E..H T.I....... .........L ..T....AFN GTA..G.GSK G--------- --TNN.QSTT LQEDP.AIQ. ....MT.E.T
36F1     .E...K.... .N....E..H T.I....... .........L ..T.H..NFN V.G.QNSTL- ---------- -----TQNST LKE.PDAIQ. ....MT.KLR
38F1     .E........ ...D..E..H T.I....... ......M..L ..T.T..N.. .TKG.D.VNN T--------- --TNT.GNDT LEEDQ.TIQ. ...EAT.E.T
39F1     .E......D. ......E..H T.I....... .........L ..T.Q.NATA TGNS------ ---------- ---------T LEE.P.AIQ. ....MT.EVR
40F1     .E......D. ......E..H T.I....... .........L ..T.H..N.N ATNGTANG-- ---------- -----LHNST LTE.P.AIQ. ....ITSEVR
41F1     .T......D. ......E..H T.I....... .........L ..T....N.. STNGTQNRTN ---------- ----DTQY.A .LE.P.EIQ. .T...T.IVR
42F1     .......... ...D..E..H T.I....... .........L ..T...KN.N TS..QN---- ---------- --------IT LQEDAKEIQ. ....MT.EVR
43F1     .-.I....D. ......E..H T.I....... .........L ..T.H.V..N ATTNGTQ--- ---------- -------S.P LKEDP.AIQ. ....MT.EVR
45F1     .E........ ...E..E..H T.I....... .........L ..T...NNTN TTNG------ ---------- ---------T LKE.T.AIQ. ....MT.EVR
46C      .A........ .D.D.....H Q.I....... .........L ..T....KEN VTRNGTV--- ---------- ------EV.D .NTMGVM... ....MT.EVR
47C      .A........ .E.D.....H Q.I....... .........L ..T....NVK VTNGTGE--- ---------- -------VKN NTM..VV... ....MT.ELR
48C      .E........ .E.D.....H Q.I....... .........L ..T.T.MNVN SSRG.D.--- ---------- -------H.G .ANMVEE... ....MT.ELR
49C      .E........ .D.D.....H Q.I....... .........L ..T.....VK SLNRT----- ---------- ---------M NETMK.D... ....AA.VLK
50C      .E........ .E.D.....H Q......... .........L ..T.S..SNI .IINSTS.S- ---------- ----NVNNTT INNTT.EI.. .T...T.E.R
51C      ......D... .E.D.....H Q......... .........L ..T....A.N RTLIQRNFT- ---------- ----TGGSTY .DSTV.EI.. ....MT.ELR
53C      .E........ .E.D.....H Q......... .........L ..T....N.N STTA..---- ---------- -------.MA NSTIM.EI.. .T..MT.EVK
54C      .E....D... .E.D.....H Q.I....... .........L ..T....N.N RTVF.D---- ---------- -------.VV ENIKKEEI.. ....MT.EVR
55C      .G........ ...D.....H Q.IV.....F .........L ..T...SNNI PT.------- ---------- ---------- -NYSD.EI.. ....MT.ELK
56C      .E........ .E.D..E..H Q.I....... .........L ..T....K.N L.N.AENV-- ---------- ------NIAN NTTIGEEV.. .T..MT.ELR
57C      .E........ .N.D..E..H Q.I....... .........L ..T.H.VNVN VTVK.R.E-- ---------- -----ITK.L .E.IR.E... ....TT.ELR
58C      .E....E.D. .E.D.....H Q.I....... .........L ..T...S..N RTR.GDHKTT DST------- -SNTS.NSSY KD.MK.EI.. ....AT.E.N
59C      .E....Y... ...D..E..H Q.I....... .........L ..T.H.S..K D.A.VNN--- ---------- -------NAT VNNSTDEI.. .T..IT.E.R
60C      .G........ .E.D.....H Q.I....... .Q.......L ..T.H.S.YS VTDPYKDL-- ---------- ------NDSI RGEIK.EI.. ....IT.ELN

                                                                          V1
                    C1
```

```
                 10         20         30         40         50         60         70         80         90         00
                                                                                                                         300
17B      DKKHQEYALF YKADVVTTKD ---------- -SNSTTSYML ISCNASVVTQ ACPKITFEPI PIHYCTPAGF VILKCNDKMF NGTGPCKNVS TVQCTHGIRP
20B      ..VQK...I. N.Y...PIDN ---------- --DNNRT... .N..T..I.. ....TS.... .......P.. AL....N.T. ......T... ........K.
21B      ..MQTQ.S.. ..L...PLET NSSVK--EAN S.QNYSD.R. ....T..I.. ....V..... .....A.... A.......K. ....S.T... ..........
22B      .RRRK..... ..T.I.SADG ---------- ---GN...TM .N..T..I.. T...V..... .......... A.......K. .......... S.........
23B      NTVRE.H... NRL...PIDN ---------- --.NNE.... .G..T..I.. ....T..... .....A.... A....G..K. ......Q... ........K.
24B      N.MQK..... .VH...L.N. ---------- ---T....T. .N..T.II.. ....VS.... .....A.... A.......K. .R........ ........K.
25B      KRVRVD..I. N.L.I.PIDN ---------- --KNS.E... .N..T..I.. ....VS.... .....A.... A....K..E. .......... ..........
26B      .RQKK..... .RL...PIDT ---------- ---DN...T. .N..T..I.. ....VS.... .....A.... A....K..K. .......... ..........
27B      S.MQK..... ..L..TPIEE ---------- --GDN..... ....T..I.. .......... .....A.... A.......Q. ......G... ........K.
28B      KGISK..... ..L.I.QIDN ---------- ----.SN... ....T..I.. .....N.... .....A.... AL......K. ......R... ..........
29B      N.IRK..... .TL.L.PIDN KTYI---DNT TNINY...T. .H.DT..I.. ....VS.... .......... A.......K. ....S..... .I......K.
30B      N.RQK..... NSL...PIDQ N--------- KTVNH..... LN..T..I.. .....S.... .......... A.......K. ......T... ..........
1B/Bbr   N.MQK..... RTLYIIQINT ---------- ------I.T. .N..T..I.. .....S.... .....A.... A.......K. ......R... ..........
2B/Bbr   ..MQK..... ..LN..PIDN ---------- -----K..T. .N..T..I.. ....VS.... .......... A..Q.K..K. ......T... ......R...
3B/Bbr   N.MQKQ.... ..L...QIDQ ---------- --TDN..... .N..T..I.. ....VS.... .......... A.......K. ......T... ..........
4B/Bbr   NEVRKD..I. NRL.L.S.VP ---------- --TDN..... .H..T..I.. .....S.Q.. .....A.... A..Q...GK. .......... ..........
5B/Bbr   N.VKR...I. .AL.I.PINA ---------- ---.SN.... TH..T..I.. .....S.Q.. .....A.... A..Q...GK. .......... ..........
6B/Bbr   GRWRK..... S.L...PIDN TS-------- AAAND..... .N.DT..I.. .....S.... .....A.... A.......K. ......T... ..........
7B/Bbr   K.VRKD..I. NRI.I.PID. NDS-----KN N.SNN..... R..DT..I.. ....V..... .........Y A.......K. .......... ........K.
8B/Bbr   N.RQK..... ..S.I.PMDN D--------- -N.NS...R. TQ..T..I.R ....TS.... .......... A.......K. ....T..... ..........
9B/Bbr   N.LQK..... .QL.LEQIDK ---------- --.DN...T. ....T..I.. .......... .....A.... A.......K. .......... ........K.
10B/Bbr  K.VRK.N.I. DSL.I.PIDE DN-------- NNETNR.... VN..T..I.. ....VS.... .....A.... A.....N.T. S......... ..........
11B/Bbr  K.MRR...I. N.L.I.PINN ---------- ---NN..... VN..T..IR. ....T..... .......... AL...K..E. ..S...T... ..........
12B/Bbr  N.VRR..... NRL.I.PINP ---------- -.DNN..... VN..T..IR. ....T..... .......... AL...K..E. ......T... ..........
13B/Bbr  NEVRKD..I. NRL.L.S.VP ---------- --TDN..... .G..T..IR. ....T..... .....A.... A.....N.T. ......T... I.......K.
14B/Bbr  N.RQK..... ..S.I.PMDN D--------- -N.NS...R. TQ..T..I.R ....TS.... .......... A.......K. ....T..... ..........
31F1     ..QLKVQ... ..L.I.PISN ---------- ---NSNE.R. .H..T.TI.. ....VSWD.. .....A...Y A.....E.N. .......... ..........
32F1     ..QQKVH... .RL.I.PING T--------- GNQNESE.R. .N..T.TI.. ....VSWD.. .....A...Y A.......S. ......R... ........K.
35F1     ..QKKVH... ..L.I.PING NG-------- SG.GSGE.R. .N..T.TI.. ....VSWD.. .....A...Y A.......K. .......... ........K.
36F1     ..QQKVH... .RL.I.PING T--------- GNQNESE.R. .N..T.TI.. ....VSWD.. .....A...Y A.......S. ......R... ........K.
38F1     ..TRKVH... .RL.I.PLNN EDK------N S.SNG.A.R. .N..T.TI.. ....TSWD.. .....A...Y A.....N.T. ......N... ........K.
39F1     ...QKVS... ..L.I.PIGN GS-------- NN.DSKE.R. .N..T.TI.. ....VSWD.. .........Y A....R..Q. .......... ........K.
40F1     ..QLKAN... ..L.I.PISN G--------- -G.GSGTNR. ....T.TI.. ....VSWD.. .....A...Y A.......K. .......... ........K.
41F1     ..QQKVH... .RL.I.PMNK S--------- SNE.GNY.R. .N..E.II.. ....VSWD.. .....A.... A.....N.T. .......... ........K.
42F1     ..QLKVH... .RL.I.PINN KS-------- SND.SIK.R. .H..T.TI.. ....VSWD.. .....A..SY A....K..K. ......R... ........K.
43F1     ..QLKVHS.. .RL.I.PINN V--------- -..GSGE.R. .N..T.TI.. ....VSWD.. .....A...Y A.....E.E. .......... ........K.
45F1     ...MKVH... .RL.I.PISN S--------- SNS.NRE.R. .N..T.TI.. ....VSWD.. .....A...Y A.......N. .......... ........K.
46C      ..TTKVH... ..L.I.QL.E ---------- -GSN.SE.R. .N..T.AI.. ....VK.D.. .....A...Y A.....N.T. ......T... ........K.
47C      ..QKKVH... .RL...PL.E DNNT----SE N..TSVE.R. .N..T.AI.. ....VK.D.. .....A...Y A.....N.T. ......N... ........K.
48C      .R.ERVH... .RL.I.PL.E ---------- ---NDGT.R. .N..T.AI.. ....VS.D.. .......... AL....N.T. ......N... ........K.
49C      N.RQKV.... .RL...PLNE REN------N A.GNYSD.I. .N..T.AI.. ....V..D.. .........Y AL....N.T. ......N... ........K.
50C      ...Q.T.... .RL.I.PLGN ---------- --E.SNN.R. .N..T.AI.. .....S.D.. .....A...Y A.....N.T. ......N... ........K.
51C      ...KKVH... .RL.I.PLDE KSNNASGSNN TVANA.E.R. .N..T.TI.. ....VS.D.. .....A...Y A.......T. ......N... ........K.
53C      .R.SKVQ... .SL...PIEN ---------- -GENNN..R. .H..T.TIA. ....VS.D.. .....A.... A.....N.T. ......N... ........K.
54C      ..RRKVH... .RL.I.PL.K NNGTS--DEE SDGTFNN.R. .N..T.AI.. ....VS.D.. .....A...Y A....K..K. ......N.I. ........K.
55C      ...KKVQ... ..I.I.PLEG NS-------- N.SDSGY.R. .N..T.AI.. ....VS.D.. .....A...Y A.....N.T. ......N... ........K.
56C      ...K.V.... ..L.I.PLE. GSN------N T..TSGN.R. .N..T.AI.. ....VS.D.. .....A.... A.....N.T. ......N... ........K.
57C      ...REA.... ..L.IAPLNS Q--------- -NEN.S..R. .N..T.AIA. ....V..D.. .........Y A.....N.T. ......N... ........K.
58C      ..IKKVH... .RL.I.PLNE S--------- -EYGKN.... .N..T.AIA. ....V..D.. .....A...Y A.....N.T. ......H... ........K.
59C      ...R.VH... .RL.I.PLS. E--------- -P.NSRD.R. .N..T.AI.. ....VS.D.. .....A...Y A.......T. ......N... ........K.
60C      ...KKVH... .RP...PLE. ---------- ---DNS..R. .N..T.AT.. ....VS.D.. .....A...Y A.I...N.T. ......H... ........K.

                           V2                                                       C2
```

```
                     10         20         30         40         50         60         70         80         90         00
                                                                                                                              400
17B       VVSTQLLLNG SLSEGDIVVR SENVTDNGKT IIVQLNESIV INCTRPNNNT RKRVT--LGP GRVYYTTGEI IGDIRKAYCN ISGIKWNNTL QQIAKKLK--
20B       .......... ....EGVII. ...I...S.. .....HKPVK ...I...... ..GIH--M.. ..TF.AR... ..N..S.... ..RA...D.. KE..E...--
21B       .......... ..A.EEV.I. ...F.N.AR. ......T.VE .......... ..SI.--I.. .AAFF-.... .....S...T .N.TQ..... K.VVT..R--
22B       .......... ..A.EEV.I. .Q.I...AR. ...H....VQ ......G... .RSIH--I.. .SAW....D. .....Q.H.. ..EAA..R.. K.VVA..R--
23B       .......... .....E.II. ...IS..A.N ......Q.V. .......... ..GI.--I.. ..AFFA..S. .....Q.H.. L.KKD.GD.. KK.EE...--
24B       .......... ..A.EEVII. ...F.N.A.. ..A...Q.V. ...V..G... ..SIH--... ..AL.A..S. .R........ LNKTH..... K..VV..S--
25B       .......... ..A....MI. ...I...A.. .......TVE .......... ..SIH--M.. ..AF.A..D. .....R.H.. ..KA...... R.V.G..R--
26B       .......... ..A.E.V.I. ...I.N.A.. .......TVE .......... .RSIH--F.. ..TL.A.... .....R.H.T .NAT...... EK.VRE.RKL
27B       .......... ..A.EGV... .K.FS..AE. .....K.PVE .......... .R.LS--F.. ..TI.A..Q. .....Q...T LNTT...... T.V.E..R--
28B       .......... ..A.EEVI.. ...F.Q.T.. ......Q.VE .K........ ..GIH--M.. .KAF.L..D. .....Q.H.. ..ET....M. K.V.E...--
29B       .......... ..A.E...I. T..ISN.A.N ......A.VA .......... .RSIN--I.. ..AFF...Q. .....Q...I LN.TI..... R.V....Q--
30B       ....H..... ..A..EV.I. ...F.N.A.N ........VE ...I..S... ..SIH--I.. ..AF.AI.G. .....Q.... LNRGN.EK.. K.VVT..G--
1B/Bbr    .......... ..A.EEV.I. .K.FS..T.. ...H..HTVK ...S..G... ..GIH--..W ..SLFA.EI. V....Q.H.. L..AE.D... R.VVE..G--
2B/Bbr    .......... ..A.EK..I. ...F.N.A.. .......TVK .D.I..S... ..SIH--..W ..AF.A.... .......... L.KTE.KKL. E...I..R--
3B/Bbr    .......... ....EEVII. ...F.N.V.. ......K..E .......... ..SIH--..W ..AF...K.. .....Q.H.. L.RVE.GT.. G...T...--
4B/Bbr    .......... ..AKE.VII. .Q.I.N.S.N ......T.VE ......G... ..GIH--..W ..AF.A..A. .........T LNKTQ.E... KYVEA..Q--
5B/Bbr    .......... ..AKE.VII. .Q.I.N.S.N ......I.VG ......G... ..GIH--..W ..AF.A.... V......H.. LNSTQ.ES.. KYVEA..Q--
6B/Bbr    .......... ..A.EEVII. ...IS..V.N ......K..A .....LH... ..GIH--..W ..ALFA.DR. V.N..Q.H.. L.RAA..D.. K..V...R--
7B/Bbr    .......... ..A.EEVII. ...I.....N .......TVK .......... ..SIH--M.W ..AF.A..A. .....Q.H.. L.RAE..K.. EK..I...--
8B/Bbr    .......... ..A.EE.I.. ...F...V.. .........Q ......S... S.GIH--..W ..AF.A.ER. .....E.H.. L.KA..ED.. .K..N...--
9B/Bbr    .......... ..A.KE.II. ...I...S.N ......A.VE .K....G... ..SIH--M.W ..AF.A..D. .....Q.H.. LNNV..S... K...G...--
10B/Bbr   .......... ..A.KEVII. ...F.N.A.. .......TVQ ...S...... ..SIH--M.W ..AF.AR.D. .....R.H.. .NEAQ..K.. G..VE...--
11B/Bbr   .......... ..A..E.II. ...I.N.... ..I....AVE .......... ..GIH--M.W .KAF.A.... ..N..Q.H.. ..EAR..... KG.VT..R--
12B/Bbr   .......... ..A.EE.II. ...I.N.A.. .......TVE .......... ..GIH--M.W ..AF.A..Q. ..N..Q.H.. .N.T...... YL..T..R--
13B/Bbr   .......... ..A.EE.MI. ...I.N.A.. .....K.A.Q .......... .RSIH--M.W ..AF.A.... V.N..Q.H.. LN.TQ.KKI. N.TVI..R--
14B/Bbr   .......... ..A.EE.I.. ...F...V.. .........Q ......S... S.GIH--M.W ..AF.A.... V.N..Q.H.T LN.TQ.KKI. N.TVI..R--
31F1      .......... ..A....II. .Q.LS..V.. ...H..Q.VP .R........ ..SIQ--... ..AF.A..N. .......H.. V.RSQ..T.. D.VKT..R--
32F1      .......... ..A....II. .Q.ISN.A.. ...H....VQ .......... ..SI.--... .QAF.A..D. .......H.. V..SQ..K.I GLVRT..E--
35F1      .......... ..A.EN.II. .Q.IS..T.N ...HF...VQ ......S... ..SIA--I.. ..AF.A.... .....R.H.. V..TQ..K.. H.VKAE..--
36F1      .......... ..A....IIK .Q.ISN.A.. ...H....VQ .......... ..SI.--... .QAF.A..D. .......H.. V..SQ..K.I GLVRT..E--
38F1      .......... ..A.E..II. .Q.IS..T.N ...H..Q.VQ .......... ..SIR--I.. .QTF.A..D. .......H.. V..EQ..K.I E.VRT..G--
39F1      .......... ..A.E..II. .Q.IS..A.I ...H....VQ .......... ..GIH--I.. .QAF.A.TD. .....Q.H.. V..TQ..K.. ERVKA...--
40F1      .......... ..A.EN.II. .Q.IS..A.. ...H....VQ .......... ..SIH--... ..AF....R. .......H.. V..TQ..K.I ERVRAA.R--
41F1      .......... ..A.E..II. .Q.IS..A.. ...H....VQ .......... ..SIH--... .KAFFA.... V......H.. V.RTQ..T.. NRVKA...--
42F1      .......... ..A.E..II. .Q.IS..A.. ...HF...VQ .......... ..SIP--M.. ..AF.A..D. .......... .N..Q..KK. REVKE...--
43F1      .......... ..A.E..II. .Q.IS..A.. ...HF...VQ .......... .RSIH--... ..AF...... .......H.. V.RSQ..K.I E.VRA...--
45F1      .......... ..A.DE.II. .Q.IS..T.. ...HF...VK .......... ..SIH--I.. .QAF.A..D. .......H.. V.EV...A.. EKVK...Q--
46C       .......... ..A.EE.II. .K.L...A.. .....K.PVL ......G... .TSIR--I.. .QTF.A..A. .....Q.H.. ...TA..K.. .KVGE..A--
47C       .......... ..A.EE.II. .K.L...A.. .....K.PVI .......... .TSIR--I.. .QT..A..D. .....Q.... ...KA..K.. .EVGE..A--
48C       .......... ..A.EE.II. .K.I...A.. ......K.VE .....F.... ..S.R--I.. .QTF.A.... ..N..Q.S.T LNETE..K.. KEVRD..R--
49C       .......... ..A..Q.II. .K.IS..T.. .......TVE .......... ..SIR--I.. .QTF.A..D. .....E.H.. ..ATA..K.. YRV.EA.A--
50C       .......... ..A..E..I. .K.L...A.. ...H..T.VR .......... ..SIR--I.. .QTF.A..D. .....Q.H.. ..RTE..R.. .EVRE..A--
51C       .......... ..A.EE.IIS .K.LSN.ADN ...H..R.VE .......... .TSIR--I.. .QAF.A..D. .....Q.H.. ..RRA..E.. EEVK.A.G--
53C       .......... ..A.EG.II. .K.I.N.A.I ...H....VE .......... ..S.R--I.. .QTF.A..D. .....Q.H.. ..KEA..R.. .LVG....--
54C       .......... ..A..E.II. .K.LA..V.. ...H.K.PVE .....F.... .ESIR--I.. .QIF.A..D. .....R.H.. ..WTA..R.. .EVRG..T--
55C       .I........ ..A.ER.II. .K.I.N.A.. .....K.PVE .......... ..SIR--I.. .QTF.A.... ..N..Q.H.. .NKAA.EKM. YGV.TE.Q--
56C       .......... ..A.EQVIIS CK.L...A.. ...H.KTPVE ......G... ..SIR--I.. .QTF.A..D. .....Q.H.. ..KTR..Q.. .EVG...A--
57C       .......... ..A..N.TI. .K.I...T.I ...H.K.PVQ .......... ..SIR--I.. .QAF.A.... .....Q.H.. ..K.A..K.. .EVG.E.A--
58C       .M........ ..A.EE.II. .K.IS..V.. ...H..TPVE .......... .TSIR--I.. .QSFFA..D. .....Q.H.. ..RAQ..D.. KKVGE..A--
59C       .......... ..A.EE.II. ...IA..A.. ......K.VE .......... ..SIR--I.. .QTF.A..D. .....Q.H.. ..KAE.EE.. .KVRN..A--
60C       .I........ ..A.KE.II. .K.I.N.VN. ...H....VE .......... .TSIR--I.. .QTF.A..D. ..N..Q.H.. ..KNA.YK.. REVS...E--

                                                                        V3
```

```
                10         20         30         40         50         60         70         80         90         00
17B      GIF------- -NKTIVF-KN ASGGDPEVVM HSFNCGGEFF YCNTTKLFDW NDTTKVFNW- -NKSWTFTEG NDTIHIPCRI KQIINRWQEV GKAMYAPPIG   500
20B      DH.K------ -...LI.-NH S......I.. .T........ ....SQ..NS TWYWNGS.-- --FNE.NLNE SE..T..... .......... .........R
21B      EQ.G------ NTT..S.-QP P......I.F .......... .....Q..NS TWNNTEMV-- --NGT.NGTT ....IL.... ...V.M.... ........LR
22B      EK.G------ -......-NS S......IEK .T........ ..D.....NS TWNNTG---- ---ESNY... .A..IL.... .......... ........LS
23B      .Y.------- -....A.-NQ S......I.. .T........ ....AE..SN TWLN------ -----KSGNN DG..TL.... .......... .........R
24B      EQ.G------ -......-NR S......I.. .......... ....SQ..NS TWFANGTR-- --.ENGTAGN .TF.TL.... .......... .........S
25B      EH.------- -....N.-TQ S......I.. F.L....... ...S.Q..NS SW.NGSWT-- --GIEGNNTS .ES.TL.... .......... .R.I.....S
26B      EL.K------ -......-NQ V......I.. .......... .....S..NS TWNNTKE--- ---WHGT.GR .N..TL..K. .......... .Q.......S
27B      KQ.G------ -.R..K.-TR S....L.IA. .T........ .....P..N. TEGSN----- -----NTQWP K.N.TLQ... ...V...... .........S
28B      EQ.E------ -....N.-TR H......IA. .......... .....Q..N. SSKLVNG--- --S.NQTSN. .R..TL.... .......... .Q.......R
29B      E.LAQA--QL V....A.AQP PP.....I.. .N........ .....N..NN GTQLLPNDT- -WQFNGSKIE .G..IL.... .......... .........S
30B      EQLKLRGQFN NST....-NQ S......I.. .T........ ...S.Q..NS SWRFNNNT.- KVVNNNTGNS ..N.TL.... ........D. .........R
1B/Bbr   EQ.N------ -KT..I.-NH S......... .......... ..D.....NS TWVINNTW-- --QRVNN.KN ESK.IL..K. .......... .........R
2B/Bbr   EQ.------- -....I.-NQ S......I.. .......E.. .....Q..NS TWNSTQLFNS TWLYN.TRNR TEN.TL.... .......... R........K
3B/Bbr   EQ.N------ -KT..I.-NQ S..K...... .....RK... ..D..Q..NI TWMYNGTW-- -QS.NINEN. .EI.TL.... .........I R..R.....K
4B/Bbr   EH.P------ -.R..Y.-NQ S....L.I.T .......... .....R..HS YWLNNTW--- ---VN.TL.I .G..TL.... .......... .........R
5B/Bbr   EH.P------ -.R..Y.-NQ S....L.IET .......... .....R..NS FWVKGTW--- ---GNSTLQN .G..IL.... .......... ..TK....LR
6B/Bbr   EQW------- -....I.-NQ SA...I.I.T .......... F....Q..NS TWLLNDTW-- -DSTGESNNT AEN.TL.... ........G. .........E
7B/Bbr   ERVN------ -KTK.I.-NQ S....S.IE. .......... .....Q..NS TWNGTH---- ----LSNSI. .E..TL.... .......... .........S
8B/Bbr   EHYN------ -.R..S.-NK S....D.I.R .......... ....SQ..N. TERNRNS--- --SDG.GSNE TE..TL.... .......... .........K
9B/Bbr   EL.------- -......-NP SA.....IE. .T........ ....SQ..NS TWNGIE---- ----NISNDT ENY.TL.... .......... .........K
10B/Bbr  EQ.------- -....I.-NQ S......I.. .TV....... ........NS TWNSSST--- --WYNKSA.. .S..IL.... .......... .........P
11B/Bbr  EK.N------ -....I.-NQ S......... FH........ ..DS.Q..NS TWLFNDT--- --RNG.DI.. .E..TL.... .......... ...I.....E
12B/Bbr  EK.N------ -....I.-NQ S......I.. FH........ ...S.Q..NS TWFNGTW.S- -TDTKGNNT. S.K.IL.... .......... .........A
13B/Bbr  EH.N------ -DR..I.-NH S......L.Q .......... .....Q..NS TWDIYGW--- ------NGTI PAN.TL.... .......... ........VS
14B/Bbr  EH.------- -DR..I.-NH S......L.Q .......... .....Q..NS TWDIYG---- ----NLNGTI PAN.TL.... .......... ........VS
31F1     SH.N------ -....I.-RP P....L.ITT .....R...L SF..NN..ND TVI------- ------NDTT TVN.TL.... .K.L.VR... EG....N...
32F1     SY.H------ -.T..K.-NS S....L.IT. .....R.... ....SW..NN TE-------- --------FN DS..TL.... ...V.M..G. .R....A..A
35F1     SH.P------ -.G..K.-NS S....L.IT. .T...R.... ....SGM.ND T--------- --------.S .EN.TFQ.C. ...V.M.... .R.K..N.VA
36F1     SY.H------ -.TN.K.-NS P....L.IT. .....R.... ....SR..NN SE-------- --------FN .S..TL.... ...V.M..G. .R....A..A
38F1     AH.P------ -....K.-EP S....L.IT. .....R.... .....R..NI T.KN------ ------IIDK .S..TL.... .....M.... .R....A..A
39F1     PH.N------ -.A..K.-NS S....L.IT. .M...R.... ....S...ND T--------- --------VS ....IF..K. .....M.... .R....A.LA
40F1     PH.P------ -....K.-NS ST...L.IT. .....R.... ....SR..ND T--------- --------KF ....VL.... R.LVRL.... .R....V..A
41F1     AH.P------ NTTI.K.-NS .....L..TT .....R.... ....SG..ND TGPSN----- ----D.RSND TEV.TL.... ...V.M.... .R....A..A
42F1     SH.------- -PVN.T.-NS SA...L.IT. .....R.... ....SA..NN ETGSN----- -----DT.GS .S..TL.... ..VV.M.... .R...TA..A
43F1     FY.P------ -.A..K.-NS S....L..T. .....R.... ....SG..ND SI-------- -------IN. TES.TL.... ...V.M.... .R....V..A
45F1     SY.P------ NRTE.R.-NS S......IT. .....RR... ....SG..NM SHNN------ -----GNDTT TV..TL.... ...V.M.... .R....A..A
46C      ER.H------ -....K.-T. H....V...N NII....G.. .F..SS..NN TYCPNNP--- --NATYNATE .S..T.Q.K. ...V.M.... .R...P...K
47C      ER.P------ -....E.-TK H....I.I.T .......... ....SG..NS TYWPNAT--- --YNA.ENSE .S..T..... ..FV.M.... .R.......R
48C      RH.P------ -.R..S.-A. H....L.ITT .....R.... ....SS..NR .I.E------ -----KI.DN .SS.T.T... ...V.M..G. .R.I.....E
49C      KY.P------ -.S..N.-TE P......ITT .....R.... ....SN..NN IYPKWNMFD- -WSNANK.NT TTN.TLR... R.F..M.... .R.......E
50C      EH.P------ -.R..K.-AK H....L.ITT .......... ....SI..ND TYWVNG---- ----TGS.N. TEN.TL.... .....M..G. .Q.I.....K
51C      KH.P------ -....T.-T. H....L.IIT .....R.... ..D..G..TE .S.EI----- -----DNGTE .S..I..... R.FV.M.... .R.I.....A
53C      EH.P------ -....Q.-TK P....L.ITT .......... ....SN..NS TYNSNC---- ----TSNRT. TEN.T..... .....M..G. .R.......K
54C      EH.P------ -....Q.-AE HA...L.ITT .....R.... ....S...NS TYWPN----- -----GTENT .I..T..... .....M.... .R.......K
55C      KH.P------ -..K.K.-T. H....L..TT .....R.... .....S..NY PNIGNSTYND TENRNSTYND TEN.T...K. ..F..M.... .R....S..E
56C      EH.P------ -.G..K.-.Q H....L.ITT .......... ..D.ST..NS IYWPHSTS-- --T.S.PGAE ...LT.Q... ...V.M.... .R.......A
57C      KH.P------ -....K.-T. S....L.IAT .....R.... ....SG..NG TY.PNS---- ----NSTET. .S..T..... .....M..G. .R.......S
58C      EH.P------ -....I.-NS SA...L.ITT .....R.... ....SG..NS TYFNGT---- ----YNS.GN SPN.TL.... ...VRM..R. .Q.......P
59C      KH.P------ -....S.-TR H....L.ITT .......... ....SS..NR TYPANST--- ---GYNNET. .S..T..... .......... .Q.......A
60C      KH.P------ -S...K.-E. S....L.ITT .....R.... ....SN..NY .YKVNS---- ---ANKD.KS .G..T..... .....M..G. .R.......A

                          C3                                    V4                                      C4
```

```
                  10         20         30         40         50         60         70         80         90         00
17B       GLIRCSSNIT GLLLTRDGG- -------NET RDNDTFRPGG GDMRDNWRSE LYKYKVVRIE PLGVAPTKAK RRVVQREKRA V-GAIGAMFL GFLGAAGSTM   600
20B       .M.N.T.... .....W...- -------.NN .T.E...... .......... .......K.. ...L...... .......... .-.T...... ..........
21B       .K.N.V.... ..I......- -----INRTK NETE...... .......... .......K.. ......PR.. .......... .-T-L..... ........N.
22B       .N.S.L.... .V.......- --------.. NAPE....Q. .NIK...G.. .......K.. ......P... ...G...... .-.-...... ........N.
23B       ...E...... ..I......- -----NNSAN NASE...... .N.K...G.. .......K.Q ...I...R.. ........K. .-T-L..... ....T.....
24B       .Y.N...... .........- ------TGNK TEPEI...RR ...K...... .......E.. ...I...... .......... .-EML..... RS.......I
25B       .QLN...... .........- ------NRTN GEAEV..... .N.K...... .......K.. ...I...... .......... .-.T...... ..........
26B       .K.N.L.... ....I....- ------V.VS NTTE...... .NIK...... .......K.. .F....PRS. ...G...... .-T-L..... ..........
27B       ...N.L.... .........- --NK--SGKD TGPEI...A. .N.KG..G.. .......K.. ...I..PR.. .......... .-.V...... ..........
28B       .Q...T.... .........- --NR--NTTD NNTEV..... .......... .......K.. ...I...R.. .......... A-.-L..L.I ..........
29B       .......... ..I......- -----YN.KS NNTE...... .......... .......K.K .......... .......... .-T-...... ..........
30B       .Q.S...S.. .........- -------.NK ESPEA..... ...KG..G.. ......KNK. ...I..P... .......... .-.T..G... ........S.
1B/Bbr    .Y.N...... .........- -------DNN NN.E....Q. .N.K...... ......IK.. .......E.. .......... I-.VL..V.. ..........
2B/Bbr    .Q........ .........- -----RD.NN .TSEI...RR ...K...... .......K.K ..K....... .......... .-.TFR.L.. R..R......
3B/Bbr    .Q........ .......SS- -----NSSDK NRPE.....K ...K...... .......K.K ..R......R .....K.... .-RT.R.... R..R.....I
4B/Bbr    .H.N.L.... ..I.A....- ------I.V. N.SE...... .......... .......K.. .......... .......... .-.TL..... ..........
5B/Bbr    .HLN.F.... .VI.A....- ------I.V. NNTE...... ..K.G..G.. .....I.KN. .......... .......... .-.T...... ..........
6B/Bbr    .......... ..I......- ------GDNN .SKE...... .G..G..G.. .......KN. ...I..PP.. ..GG...... .-.T..G... ....T.....
7B/Bbr    .Q.S...... ..I.....S- ------NDNS ST.E...... .N.K...... .......E.. .V.L...... .......... .-.TL..... ....T.....
8B/Bbr    .N.S...... .........- ----TNLTGS NGTE....Q. .N.K...... .......... .V.I...E.. .......... .-.TL..... ..........
9B/Bbr    .N.S.T.... ..I......- ------N..S NETE...... .......... .......K.. ...I...... .......... .-.TL..... ..........
10B/Bbr   .H.N.L.... .........- -------.NN SSSE...... .......... .......... .........R .......... .-.T...... ..........
11B/Bbr   .Q...T.... .........- ---------N SGTE...... .......... .......K.. ...I...... .......... .-.T.E.... ..........
12B/Bbr   .Q.K.T.... ........-- --NGN-R... EPSE...... ....G..... .....I.KN. ...I..P... .......... .-.T...V.. ..........
13B/Bbr   ...N.T.... .........- ------I..N NISE...... .......... .......K.. ...I..S..Q .......... .-.TL..... ..........
14B/Bbr   ...N.L.... .........- ------I..N NISE...... .......... .......K.. ...I..S..Q .......... .-.TL..... ..........
31F1      .K.T.T.I.. .IV...E..- ------N.RG NN.E...... .N.K...... .......E.. .......... .Q..K..... .-.-M..L.. ..........
32F1      .N.T.N.... .........- --------QN NT.E...... .E........ .......E.. .......E.. ....K..... .-.-...V.. ..........
35F1      .N.T.N.... ..I......- -------.NS TG.E...... .N.....G.. .......E.. ...I..PQ.. .Q..A..... G-.-K..F.. ...S......
36F1      .N.T.N.... .........- --------QN NT.E...... .E........ .......E.. .......E.. .......... .-.-...V.. ..........
38F1      .S.T.N.... ..I......- -----SC.D. NGTEI..... .N.K...... .......E.. ...I...T.. .Q..K.D... .-.-...VL. ..........
39F1      .NLT.R.... ......E..- ---------N KE.E...... .N.K...G.. .......EN. ...I..P... .Q.GT..... .-.-KA.VIF ..........
40F1      .S.T.N.T.. .........- -----QH... NKTEI..... .N.K...... .......E.. .......G.R .Q..K..... .V.-...VLI ..........
41F1      .N.T...... .........- -----NN... NKTEI..... .N.K...... .......E.. ...I...... .Q........ .-.-M..L.. ..........
42F1      .S.T.N.... .........- -------Y.N NQTE...... .N.K...... .......E.Q .......R.. .P..R..... AV.-...... ..........
43F1      .N.T.I.... .........- -----LN.D. NQTE...... .N.K...... .......E.. ......PR.. .Q..K..... .-.-...L.. ........N.
45F1      .S.T.K.... ........S- --SN--NSTH NNTE...... KNIK...... ......IE.. ...I...... ....T..... .-.-...L.. K........I
46C       .P.T.A.... .........- -------ANS NNTE...... ...K...... .......E.K ...I...... ....E..... .-.-...V.. ..........
47C       .P.N.T.... ...I.....- -------INS S.TEI..... .......... .......E.K ...I...E.. ....E..... .-.-...V.. ........I.
48C       .V.T.R.... .........- -------.TN NTTE...... .......... .......E.K ...I...... ....E..... .-.-...V.. ..........
49C       .I.T.R.... .........R GDT-N-GT.. NKTE...... .......... .......E.K .......... ....E..... .-.-...V.. ..........
50C       .R.T.K.... .I.......- -----ET... NATEV..... .......... .......E.K ...I...R.. ....E..... A---L..... ..........
51C       .N.T.R.... .........- ----EGNETQ N.TE...... .N.K...... .......E.K ...I..P... ....E..... A---L.GV.. ..........
53C       .Q.T.R.... ..V.E....- --------TN KTEE..W.T. .......... .......EVR ...I...R.. ....E....D L-.-L..V.. ..........
54C       .I.T.R.... .........- -----NGDF. DSPE...... .......... .......E.K ...I...... ....E..... .-.-...V.. ..........
55C       .V.T.K.... .F.......- --------K. TK.E....E. .N........ .......E.K ...I...... ....E..... G---...V.. ..........
56C       .N.T.N.S.. .........E NR----TEKG TKEEE..... .......... .......E.K .F.I..P... ....E...K. A---L..V.. ........N.
57C       .I.T.R.... .I.......- -------DSN N.TEI...T. .......... .......E.K .V.I..P... ....E....G I-.-...V.. ..........
58C       .I.T.R.... .........- -------PDN NRTE...... .......... .......E.K ...I...... ....E..... .-.-L..... ..........
59C       .I.T.R.... .........- -------.T. NKTEI...E. .N........ .......E.K ...I...... ....E..... .-.-...V.. ..........
60C       .I.S.N.S.. .........- ------N.NN T.TEI..... .N........ .......E.K ...I...... ....G..... A-.-...V.. ..........

                         V5                          C5        (gp120 end)                  gp41  Fusion peptide
```

```
                   10         20         30         40         50         60         70         80         90         00
17B        GAAAVTLTVQ ARQLLSGIVQ QQNNLLRAIE AQQHLLQLTV WGIKQLQARV LAVERYLRDQ QLLGIWGCSG KIICTTAVPW NTSWS----N KSREDIWENM   700
20B        ...S...... ..L....... .......... ....M..... .......... .......... .......... .L........ .....TRWK. R.LS......
21B        ...SI..... .......... .......... .......... .......... .......... .......... .L........ .E...----. R.LNY..D..
22B        ...SL..... ..L....... .......... .......... .......... ....S..... .......... .L........ .A...----. .TLNQ..D..
23B        ...S...... .......... ..S...K... .......... .......... .......EV. .........E .L....T... .I...----. R.QKE..K.I
24B        ...S.A.... .......... ..S...K... ....M..... .......... .......K.. .......... .L..P..... .A...----. ..LNE.....
25B        ...SI..... ..L....... .......... ....M..... .......... .......... .......... .L....S... .....----S ..LDT..N..
26B        ...SMG.... .K........ ....F..... ....M..... .......... .......K.. .......... .L........ .....----. ..LDQ.....
27B        ....M..... T......... ......K... .......... .......... .....F.Q.. .......... .L....T... .....----. ..V.M..Q..
28B        ...S...... .......... .......... .......... .......... .......K.. .......... .L........ .....----. ..L.K..N..
29B        ...SL..... .....T.... .......... .......... .......... .......Q.. .......... .L........ .A...----. R.LDT..N..
30B        ...SL..... ..L....... .......... .......... .......... .......... .......... .L....T... .N...----. ..L.K..N..
1B/Bbr     ...SI..... .......... ..S...Q... .......... .......... ..L....... ....L..... .L....T... .A...----. .TLK......
2B/Bbr     ...SM..... ......S... .......... .......... .......... .......K.. ...R.....R .L........ .A...----. .TM.E.....
3B/Bbr     ...SMA.... ..L....... .......... .......... .......... .......K.. .......... .L........ .A...----. .TLDK..N..
4B/Bbr     ....T..... ..L....... .......... ....M..... .......... .......... .......... .L....T... .....----. ..L.Y..N..
5B/Bbr     ...S...... ..L....... ..S....... .......... .......... .....F.K.. .......... .L....T... .....----. ..LDY..N..
6B/Bbr     .P.SI..... ..LV.F.... ......K... .......... .......... .......Q.. .......... .L....S... .A...----. .AYDH..G..
7B/Bbr     ...SL..... ....M..... .......... ....M..... .......... .......... .......... .L........ .....----. .YLSY..N..
8B/Bbr     ...SM..... .......... ......K... ..H....... .......... ....S..... .......... .L........ .A...----. ..K.E..G..
9B/Bbr     ...SI..... ...V...... .......... M......... .......... .......Q.. R...L..... .H..P..... .A...----. R.LNN.....
10B/Bbr    ...SI..... ..L....... .......... ....M..... .......... .......... .......... .L....S... .S...----. ..LDA..D..
11B/Bbr    ...S...... ..L....... ......K... ....M..... .......... .......... .......... .L........ .S...----. .NLST..D..
12B/Bbr    .......... ..L....... ......K... ....M..... .......... .......... .......... .L....T... .S...----. ..VNY..D..
13B/Bbr    ....A..... ..L....... .......... ....M..... .......... .......... .......... .L........ .....----. ..YDH.....
14B/Bbr    ....T..... .......... ......K... ....M..... .......... .......... .......... .L........ .....----. .AYDH..G..
31F1       ...SM..... .......... .......... .......... .......... .......K.. ....L..... .L....N... .S...----. ..Q.E..N..
32F1       ...SM..... .......... .......... .......... .......... .......K.. ....L..... .L....N... .S...----. ..Q.Q..Q.I
35F1       ...SI..... .......... ......KV.. ..H......F .......... .......K.. .......... .L....N... .ST..----. R.HDY..G..
36F1       ...SM..... .......... .......... .......... .......... .......K.. ....L..... .L....N... .S...----. ..Q.Q..Q..
38F1       ...S.A.... .......... .......... ....M..... .......... .....F.... ....L..... .L....N... .S...----. ..YNM..Q..
39F1       ..GSL..... ...I.F.... ......K... .......... .......... .......K.. .R..L..... .L....S... .S...----. ..LDQ.....
40F1       ...S...... .......... ......K..D ....M..... .......... .......K.. ....L..... .L....N... .S...----. .TQTE..G..
41F1       ...S...... .......... ..S....... ....M..... .......... .......Q.. R...L..... .L....N... .S...----. .TQTE..G..
42F1       ...SIA.... .......... .......... .......... .........I .......K.. ....L..... .L....N.L. .S...----. R.HGE.....
43F1       ..GSM..... .......... .......... .......... .........I .......K.. ....L..... .L....T... .S...----. R.Q....N.L
45F1       ...S...... .......... .......... ....M..... .........I .......K.. ...RL....R .L....N... .S...----. R.Q....N.L
46C        ...SI..... .....F.... ..S....... ....M..... .......T.. ..I....K.. .......... .L........ .S...----. ..LG...D..
47C        ...SL..... .....F.... ..T....... ....M..... .......T.. ..I....K.. .......... .L........ .S...----. R.KDY.....
48C        ...SI..... .......... ..S....... ....M..... .......T.. ..I....K.. .......... .L........ .S...----. ..Q....D..
49C        ...SI..... .......... ..S....... ....M..... .......T.. ..I....K.. .......... .L....N... .A...----. ..Q.A..D..
50C        ...SIA.... .......... ..S....... ....M..... .......T.. ..I....K.. .......... .L........ .S...----. R.Q.E.....
51C        ...SMM..A. .....F.... ..S......G ....M..PPF .....F...F .VI....K.. .......... .L..P..... .A...----. ..Q.E..N..
53C        ...SI..... .......... ..S....... ....M..... .......... ..I....... .......... .L........ .S...----. R.Q.......
54C        ...SI..... .......... ..S....... ....M..... .......T.. ..I....K.. .......... .L........ .S...----. ..L....D..
55C        ...SI..... .......... ..S....... ....M..... .......T.. R.I....K.. .......... .L........ .A...----. ..QQ...D..
56C        ...S.A.... .......... ..S...K... ....M..... .......... ..I....K.. .......... .L........ .S...----. ..H.E..N..
57C        ...SI..... .....F.... ..S....... ....M..... .......... ..L....Q.. .......... .L.....L.. .S...----. R.QK...D..
58C        ...SI....H .......... ..S....... ....M..... .......T.. ..I....Q.. R......... .H........ .S...----. ..LGE..N..
59C        ...SI..... .......... ..S....... ....M..... .......T.. ..I....Q.. .......... .L........ .S...----. ..Q....N..
60C        ...SMM.... .......... ..S....... ....M..... .......T.. ..I....K.. R......... .L....T... .S...----. R.Q.E..D..
```

gp41

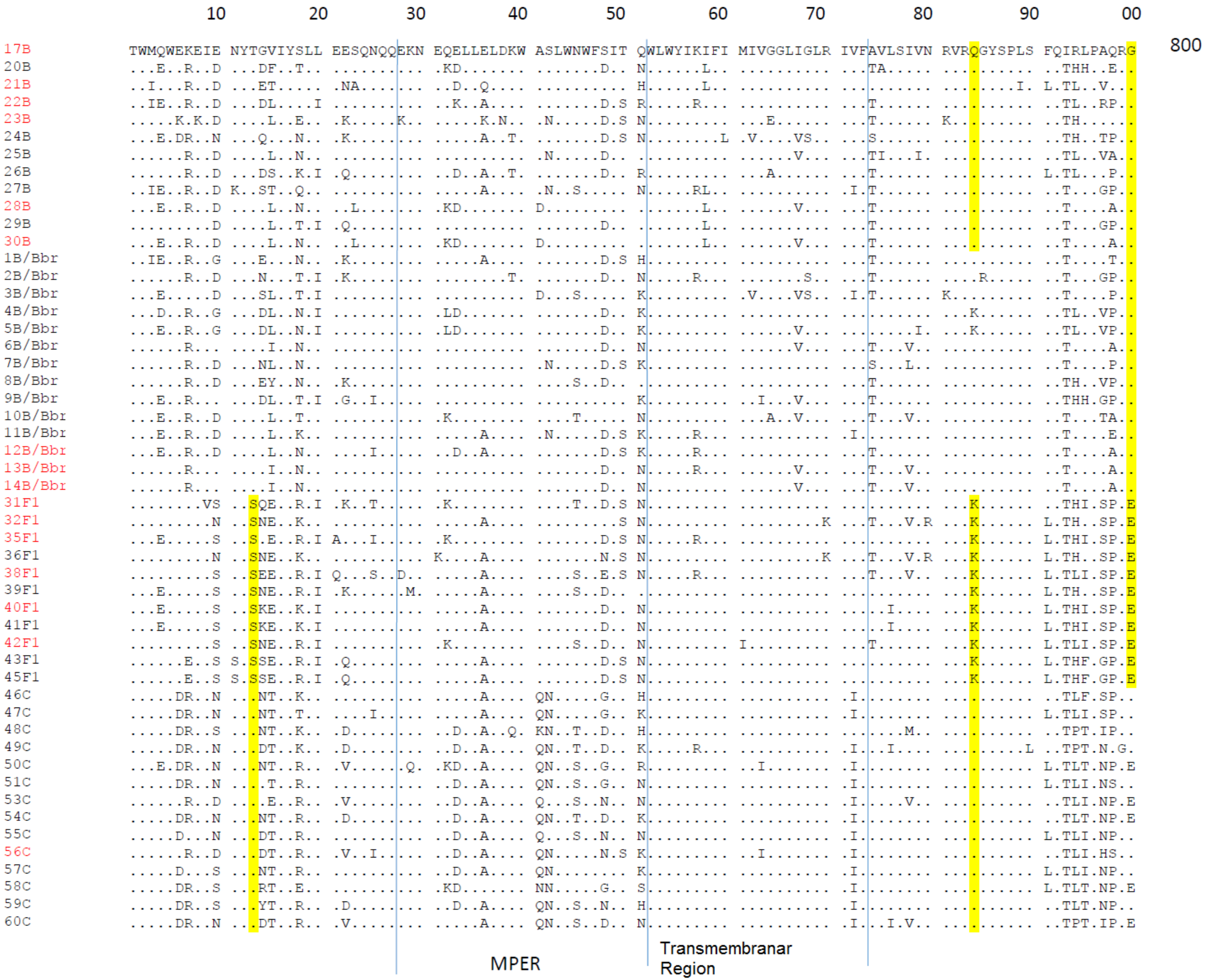

10 20 30 40 50 60 70 80 90 00
800
17B TWMQWEKEIE NYTGVIYSLL EESQNQQEKN EQELLELDKW ASLWNWFSIT QWLWYIKIFI MIVGGLIGLR IVFAVLSIVN RVRQGYSPLS FQIRLPAQRG
20B
21B
22B
23B
24B
25B
26B
27B
28B
29B
30B
1B/Bbr
2B/Bbr
3B/Bbr
4B/Bbr
5B/Bbr
6B/Bbr
7B/Bbr
8B/Bbr
9B/Bbr
10B/Bbr
11B/Bbr
12B/Bbr
13B/Bbr
14B/Bbr
31F1
32F1
35F1
36F1
38F1
39F1
40F1
41F1
42F1
43F1
45F1
46C
47C
48C
49C
50C
51C
53C
54C
55C
56C
57C
58C
59C
60C
MPER
Transmembranar Region

```
                   10         20         30         40         50         60         70         80         90         00
17B       HERPGGIEEE GGERDRDRSG PLVNGFLELI WDDLLSLCLF SYRRLRDLLL IVARIVELLG R-------RG WEALKYWWNL LLYWIQELKN SAVSLLNATA   900
20B       PD....T... .........N R..H...A.V ....R..VI. ...L...F.. ......G... .-------.. ..V.....S. .Q..S....S .....F..I.
21B       PG..E.T... ..D.N..T.S R..H...A.. ....R..... ..H....... .......... .-------.. .......... .Q..S..... .....F....
22B       PD..E.T... ..G....T.S Q.AT...AI. ....R..T.. ..HL...... SA..V.G... .-------.. .......... .Q..S..... ........I.
23B       PD..E.T... .........T R..T..FA.. ....R..V.. ..H....... .A........ .-------.. ......L... .......... .......TI.
24B       PD..RET... ..D.N..S.E R.AT...A.. .V..R..F.W ..HQ....I. VA.K..G... .-------.. ......L... .Q..S..... ..........
25B       PD..E.TG.. .....SG.FN Q.AP...A.V .V..RN.F.. ..HH...... .T..SL.... .-------.. ......L... .G..S....S ..I..F....
26B       PD..E..... ....N....T R......A.. ....R...I. ..H....... .......... H-------.. ......C... .Q..S..... ..........
27B       PDG.E..... ....N.S..E R..A..FA.. ....R..... ..H....... ..T....... .-------.. G.I....... FQ..S..... .....F..IV
28B       PD..E..... .........N R..T...P.. ....R..... ..H....... .A.....I.. .-------.. .......... .Q..S....S ..IN.F..I.
29B       PD..E..... .........T R..T...P.. ....RN.... ..HH...... .AT...G... .-------.. .......... .Q..S..... ...N.FGTI.
30B       PD..E..... .........N R..T...P.. ....R..... ..H....... .A.....I.. .-------.. .......... .Q..S....S ..IN.F..I.
1B/Bbr    PD..E..... .......... G..T...V.. .V..RN.... L..H...... .TT....... .--------. .......... .Q........ ..I..V.TI.
2B/Bbr    PDK.ER.... .R.......R R..TR..A.. ....R..... ..H....... ...KVM.... .-------.R .......... .Q..S..I.. ..........
3B/Bbr    PD..A.T.KK S........R R..TR..A.. ....R..... ..HH...... .A.......R .-------KK .......... .Q..C..... ..........
4B/Bbr    PD..E..... ......A... R.....G... ....W..LS. ..H....... .......... .--------. .......... .Q..S..... ..I..F..L.
5B/Bbr    PD..E..... .......... R.....G... ....W..LS. ..H....... ..T....... .--------. ..V....... .Q..S..... ..I.......
6B/Bbr    PD..E..... ..D..SP..R R..D...A.V ....R..... ...H...... .......... .-------.A ......G... ....S..... .....F..L.
7B/Bbr    PD...E.G.. ..D....S.E R.AT...A.. .V..R..F.C ..HH...S.. .AT....... .--------. .......... .Q..S..... ..........
8B/Bbr    PD........ ....G....E R..D...A.. ....RN.... ..H..S.... ..T....... .--------. .......... .Q..S....S ..........
9B/Bbr    PD..E.T... .........V R..H...A.V ....RN.... ..H..T..I. ..R....... .--------. ......L... .Q..S..... .......TI.
10B/Bbr   PD..E..G.. ...S..GG.T R..H...A.V ....R...V. ..H....... ..T....... .--------. .......... .Q..S...R. .....FD.I.
11B/Bbr   PD..E..G.. ..DK.....R R..H...A.V ....R..... ..HH...... ....T..... .--------. ....R.G... .Q..S..I.. ..........
12B/Bbr   PD..E..G.. ...N.....N R..H...A.V ....R..... ..HN...... ....T..... .--------. ......G... .Q..S..... ..........
13B/Bbr   PD..E..... ..D...P..R R..D...A.V ....R..... ...H...... .......... .--------. ......G... ....S..... .....F..L.
14B/Bbr   PD..E..... ..D..SP..R R..D...A.V ....R..... ...H...... .......... .-------.A ......G... ....S..... ........L.
31F1      PD..E....G ...Q.K...V R.....FA.V .E..RN.... ...H...FI. .A....D--- ----RGLR.. .....HLG.. V...S..... ..I...DT..
32F1      PD..EE...G ...QG....V R......S.V ....RN.... ...H...FI. .A..T.D--- ----RGLKW. .Q....LG.. TQ..L.K... ..I....T..
35F1      PD.LEE...G ...QG....V R.....FA.V ....RN.... ...H...FI. .A....D--- ----RGLR.. ......LG.I .Q..GL.... ..I....T..
36F1      PD..EE...G ...QG....V R......S.V .N..RN.... ...H...FI. .A..T.D--- ----RGLRW. .Q....LG.. TQ..L.K... ..I....T..
38F1      PG..E....G ...QG....V R.....FA.V ....RN.... ...H....I. ......D--- ----RGLK.. ..V..LLG.. T...GR.... ..I....TV.
39F1      LD..E....G ...QGK...V R....L.A.. ....RN.... ...H...FI. .A....N--- ----RGLR.. ......LG.. A...S..... ..I.......
40F1      PD..D....G ...Q.K...V R......S.V ....RN.... ...H...FI. .A..T.D--- ----KGLK.. ......LG.. TQ..G..... ..I....TI.
41F1      PD..D....G ...Q.K...V R......S.V ....RN.... ...H...FI. .A..T.D--- ----KGLK.. ......LG.. TQ..G..... ..I....TI.
42F1      PD..E....G D..QGKG..V R..H...A.V .T..RN.... ...L...FI. .A....D--- ----RGLR.. .....LLG.. AQ..S..... ..I....T..
43F1      PD..E....G ...QGK...V R......A.A ....WN.... ...H...FI. VA....D--- ----RGLR.. .....LLG.. TQ..S..... ..I....TI.
45F1      PD..E....G ...QGK...V R......A.A ....WN.... ...H...FI. VA....D--- ----RGLR.. .....LLG.. TQ..S..... ..I....TI.
46C       PD........ ...Q.KGK.I R..S...A.V ....R..... ..H.....T. .A..AL.... .SSLRGLQ.. ..I...LGS. VQ..GL...K ......DT..
47C       P......... ...Q.KGS.I R..S...A.V ....RN.... ..HQ....T. .AT.AL.... .SSLRGLQ.. ..I...LGS. VQ..SL...K ..I...DT..
48C       PD.L...... ...Q.....I R..S...A.A ....R..... ..H.....I. .A..A..... .SSLRGLQ.. ..I...LGS. VQ..GL...K ..I...DT..
49C       PD.L...... ...K.....I R..S...A.A ....R..... ..H.....IS .A..A..... HSSLRGLQ.. ..I...LGS. VQ..GL...K ..IG.FDTI.
50C       PD........ ...QG....I R..S...P.F ....R..... ..H.....IS .A..AA.... .SSLKGLQ.. ..I...LGS. VQ..GL...R ..I..FDTI.
51C       QD..E..D.. ...Q.....I R..S...A.A ....R..... ..H.....T. .A..A..... .SILKGLQ.. ..L...LGS. VQ..GL...R ..I..FDTI.
53C       PG.L...... ...Q.K...I R..S...A.A ....R..... ..H.....I. .A..A..... .SSLRGLQ.. ..I...LGS. VQ..SL...K ..I...DTI.
54C       PD.L...... ...Q.....I R..S...A.A ....R..... ..H.....I. .A..A..... HSSLRGLQ.. ..I...LGS. VQ..GL.I.K ..I...DTI.
55C       PD.L...... ...Q..A..I R..S..FS.A ....R..... ........I. .AT.A..... HSSLRGLQ.. ..I...LGS. VQ..GI...K ..I...DTL.
56C       PD.L.E.... ...Q.KE..I R..S..FS.A ....R..... ..H.....V. .AT.T..... .SSLRGLQ.. ..I...LGS. VQ..GL...R N.IR..DT..
57C       PD.L...K.. ...Q.....I R..S...A.A ....R..... ..H.....I. .A..A..... .SSLRGLQ.. ..I...LGS. AQ..GL...R .VI..FDTV.
58C       PD.L...... ...Q.K...I R..S...A.T ....R..... I.H.....I. .A..A..... .SSLRGLQ.. ..I...LKS. VQ..GL...K ..I...DTI.
59C       PD.L...... ...Q.....I R..D...A.A ....RN.... C.H.....I. .AT.A..... .SSLKGLQ.. ..I...LGS. VQ..GL...K ..I...DTI.
60C       PD.L...... ...Q.....I R..S...A.A ....R..... ..H....... .T..A..... .SSLRGLQ.. ..I...LG.. VQ..SL...R ..IN..DTI.
```

Citoplasmic Tail (gp41)

```
                10         20         30
17B       IAVAEGTDRV LEILQRIGRA ILHIPTRIRQ GFERALL   937
20B       .........I I.AV...... .....R.... .L.....
21B       .........I I..A...F.. F....R.... .L....Q
22B       ...G...... I.AI...... F.N..R.... .......
23B       VV........ I.VV.G...G ..N..R.... .L..S..
24B       .....R...I V..I.KV..G .....R.... SL.....
25B       VT.......I I.LA....IG L....R.... .L.....
26B       .......... I.VA...VT. F.NV.R.... .L.....
27B       .P.......I I.TI...C.. .....R.T.. .L.....
28B       .......... K.VV.T.W.. F....R.... .A..F..
29B       .........I I.AG..LC.. .....R.... .L..L..
30B       .......... K.VV.T.W.. F....R.... .A..F..
1B/Bbr    .......... I.AA..F..G .....R.... .L.....
2B/Bbr    .....R..K. ..VA..VS.S .....R.... .L.KI..
3B/Bbr    V....R..K. I.AV...S.G .....R.... .L.....
4B/Bbr    VT........ I.VV....N. ..NV.R.... .L.....
5B/Bbr    .V.......I I.AVL..C.. ...V...... .L.....
6B/Bbr    VT........ I.VV....N. ..NV.R.... .L.....
7B/Bbr    .......... I.VV.....G .....R.... .L.....
8B/Bbr    .........I I.VI...C.. .......... .L.....
9B/Bbr    .........I IGVA.....G .I...R.... .L..I..
10B/Bbr   .......... I.VA...... .......... .L....Q
11B/Bbr   .......... I.LA..L..G .......... .L.....
12B/Bbr   .......... I.LA..L..G .......... .L.....
13B/Bbr   VT........ I.VV....N. ..NV.R.... .L.....
14B/Bbr   VT........ I.VV....N. ..NV.R.... .L.....
31F1      .V........ I.A...A... ..N..R.... .L.....
32F1      .V........ I.AV..A... V.N..R.... .L....Q
35F1      .V........ ..V......G .....R.... .L..S..
36F1      .V........ I.AV..A... V.N..R.... .L....Q
38F1      .V.......I I.A...A... V.N..R.... .L.....
39F1      .V.......I ..A...A... .....R.... .L.....
40F1      .V........ I.A...A... ..N..R.... .L.G...
41F1      .V........ I.A...A... ..N..R.... .L.G...
42F1      .V...W.... ..A...V... V.N..R.... .L.....
43F1      .V........ I.A...A... V.N..R.... .L..S..
45F1      .V........ I.A...A... V.N..R.... .L..S..
46C       .........I I..I...C.. .CE..R.... ...A..Q
47C       .........I I......C.G .CE..R.... ...A..Q
48C       .V.......I I.V..G.... .YN..R.... ...A..Q
49C       .V.......I I.LV...W.. .CN....... ...A..Q
50C       ....GR...I I.LI.G.W.. .CS..R.... .L.A..Q
51C       .........I I.GV...... .YN..R.... ...A..Q
53C       .........I I.VI...WG. .CNV.R.... ...A..Q
54C       .........I I.VI.G.W.. .CN..R.... ...A..Q
55C       .........I I.AI..FW.. .CN..R.... ...A..Q
56C       .........I I.LV...W.G .RNV.R.... ...A..Q
57C       .........I I.VV...W.. .CN..R.... ...A..Q
58C       .........I I.VI.G.W.. .CNV.R.... ...A..Q
59C       .........I I..I.G.... .Y...R.... ...A..Q
60C       ...G.....I ID.VRG.C.. .CN..R.... ...A..Q
```

S1 Fig. Alignment of 51 HIV-1 envelope amino acid sequences according to B, B/Bbr F1 and C subtypes

The HIV-1 subtype-specific signatures are highlighted in yellow. Samples with high neutralization potency are noted in red.
