# Supplementary material for: Cross-Neutralizing Antibodies in HIV-1 Individuals Infected by Subtypes B, F1, C or the B/Bbr Variant in Relation to the Genetics and Biochemical Characteristics of the env Gene
Source: PLoS One. 2016 Dec 9;11(12):e0167690. doi: 10.1371/journal.pone.0167690 (PMC5147934; doi:10.1371/journal.pone.0167690)
Supplement: S2 Fig — (PDF) [file pone.0167690.s002.pdf]

S2 Fig: Alignment of *env*-psVs in relation to the HXB2 reference virus.

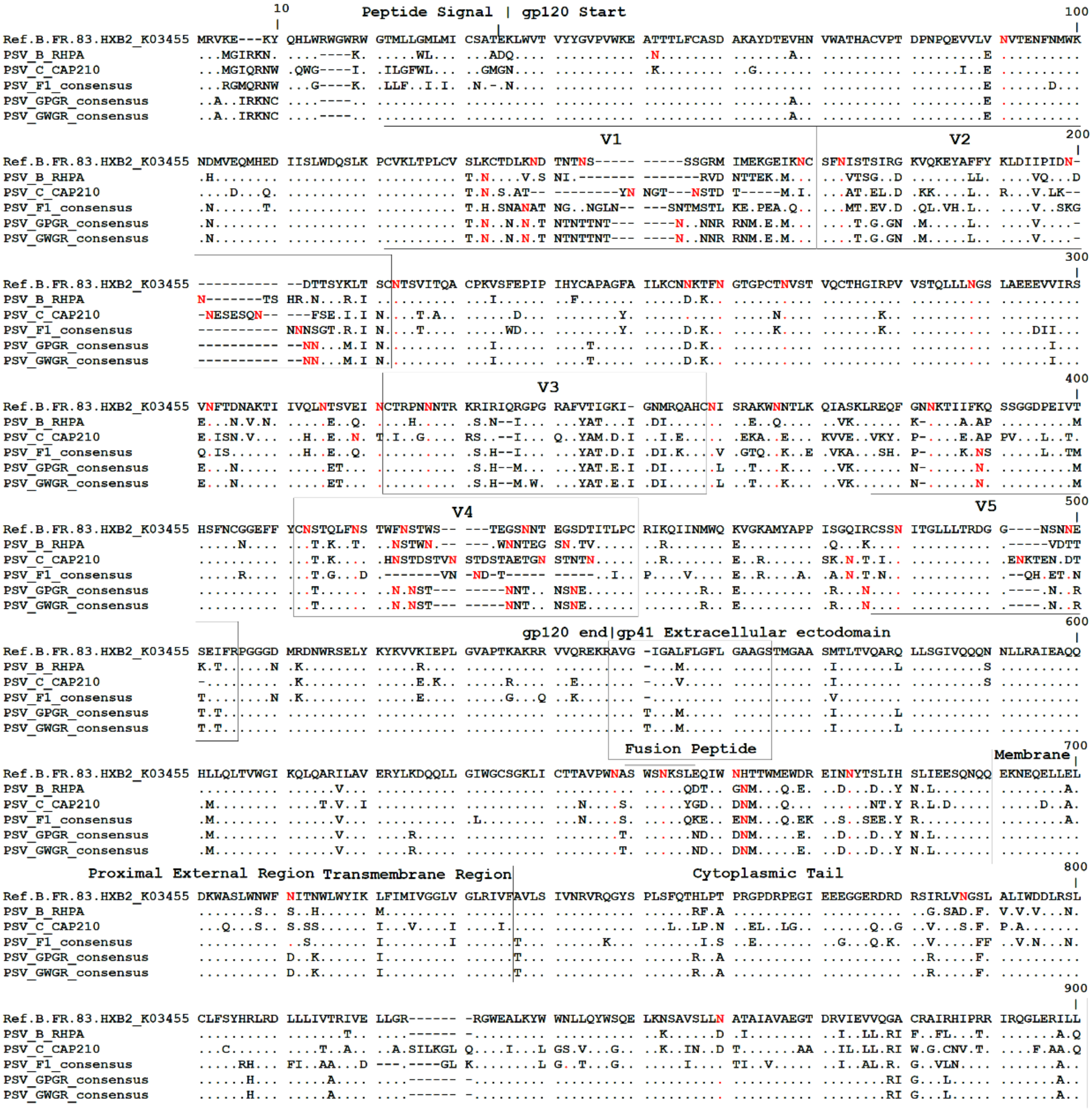

Nucleotide sequences of HIV samples from the *env* gene were translated, aligned, and produced a consensus sequence for subsubtype F1 and B/Bbr to produce the psVF1 and psVGWGR, respectively. psVCap210 and psVRhpa were obtained from nucleotide sequence AY835447.1, whereas psVCap 210 was obtained from FJ443315.1. All psVs were aligned with the HXB2 reference. Dots indicate sequence identity, whereas dashes represent gaps introduced to optimize alignments.
