# Supplementary material for: Cross-Neutralizing Antibodies in HIV-1 Individuals Infected by Subtypes B, F1, C or the B/Bbr Variant in Relation to the Genetics and Biochemical Characteristics of the env Gene
Source: PLoS One. 2016 Dec 9;11(12):e0167690. doi: 10.1371/journal.pone.0167690 (PMC5147934; doi:10.1371/journal.pone.0167690)
Supplement: S1 Table — (DOCX) [file pone.0167690.s003.docx]

**S1 Table: Plasma samples from individuals infected with the different HIV-1 subtypes** **exhibit antibodies activity profiles against distinct pseudoviruses.**

| **Sample (subtype)** | **Plasma neutralizing ID_50_ titers against *env* psV** | | | | | **GMT** |
| --- | --- | --- | --- | --- | --- | --- |
|  | **psV GWGR** | **psV Cap210** | **psV F1** | **psV GPGR** | **psV pRhpa** |  |
| 17 (B) | 6250 | 43740 | 43740 | 6250 | 43740 | 20085 |
| 23 (B) | 43740 | 13898 | 9739 | 3704 | 43740 | 15717 |
| 22 (B) | 6250 | 31075 | 12417 | 6250 | 5813 | 9739 |
| 14 (B/Bbr) | 6250 | 5260 | 11589 | 3984 | 20866 | 7946 |
| 13 (B/Bbr) | 6000 | 2217 | 12824 | 4525 | 17893 | 6731 |
| 32 (F1) | 6250 | 2382 | 6372 | 3953 | 8284 | 4994 |
| 28 (B) | 6250 | 1462 | 1926 | 6250 | 12220 | 4224 |
| 31 (F1) | 56180 | 1430 | 6631 | 2273 | 1000 | 4136 |
| 42 (F1) | 6250 | 10844 | 3215 | 3623 | 1000 | 3797 |
| 30 (B) | 2545 | 2953 | 1940 | 2618 | 4488 | 2797 |
| 12 (B/Bbr) | 13106 | 2104 | 746 | 4854 | 1134 | 2575 |
| 35 (F1) | 5025 | 3486 | 11252 | 60 | 2182 | 1916 |
| 21 (B) | 3472 | 8492 | 30 | 4545 | 5728 | 1873 |
| 40 (F1) | 20 | 24450 | 41283 | 20 | 43740 | 1776 |
| 56 (C) | 2778 | 523 | 3007 | 3378 | 185 | 1222 |
| 38 (F1) | 70423 | 273 | 3003 | 4000 | 10 | 1182 |
| 27 (B) | 3472 | 165 | 5420 | 4545 | 44 | 909 |
| 26 (B) | 16000 | 702 | 109 | 276 | 1562 | 880 |
| 24 (B) | 1055 | 356 | 97 | 730 | 2061 | 559 |
| 41 (F1) | 119 | 1726 | 103 | 1444 | 1388 | 531 |
| 29 (B) | 1250 | 197 | 2313 | 258 | 151 | 467 |
| 58 (C) | 207 | 5691 | 749 | 71 | 115 | 373 |
| 55 (C) | 6250 | 1073 | 47 | 237 | 61 | 340 |
| 39 (F1) | 98 | 7636 | 42 | 33 | 1397 | 271 |
| 60 (C) | 847 | 2626 | 109 | 55 | 40 | 222 |
| 25 (B) | 352 | 2104 | 52 | 256 | 10 | 158 |
| 53 (C) | 3831 | 1007 | 96 | 20 | 10 | 149 |
| 54 (C) | 3497 | 122 | 58 | 116 | 20 | 142 |
| 20 (B) | 110 | 20 | 83 | 43 | 4777 | 130 |
| 59 (C) | 495 | 586 | 57 | 64 | 25 | 121 |
| 43 (F1) | 305 | 1602 | 10 | 115 | 37 | 116 |
| 45 (F1) | 280 | 68 | 33 | 253 | 126 | 115 |
| 48 (C) | 621 | 55 | 1945 | 20 | 10 | 106 |
| 49 (C) | 293 | 547 | 84 | 94 | 10 | 105 |
| 36 (F1) | 85 | 20 | 216 | 363 | 88 | 103 |
| 6 (B/Bbr) | 168 | 865 | 10 | 92 | 59 | 95 |
| 9 (B/Bbr) | 12870 | 10 | 10 | 100 | 37 | 86 |
| 7 (B/Bbr) | 83 | 20 | 1654 | 63 | 20 | 81 |
| 50 (C) | 2500 | 10 | 95 | 38 | 20 | 71 |
| 10 (B/Bbr) | 1044 | 324 | 10 | 52 | 10 | 71 |
| 8 (B/Bbr) | 341 | 30 | 118 | 57 | 20 | 67 |
| 11 (B/Bbr) | 134 | 430 | 10 | 228 | 10 | 67 |
| 46 (C) | 2932 | 36 | 48 | 25 | 10 | 66 |
| 57 (C) | 138 | 546 | 20 | 20 | 20 | 57 |
| 2 (B/Bbr) | 104 | 407 | 10 | 83 | 10 | 51 |
| 3 (B/Bbr) | 39 | 818 | 10 | 28 | 22 | 46 |
| 47 (C) | 350 | 10 | 119 | 45 | 10 | 45 |
| 51 (C) | 20 | 52 | 110 | 20 | 45 | 40 |
| 5 (B/Bbr) | 67 | 81 | 10 | 10 | 54 | 31 |
| 4 (B/Bbr) | 20 | 488 | 10 | 20 | 10 | 29 |
| 1 (B/Bbr) | 10 | 169 | 33 | 32 | 10 | 28 |
| GMS | 886 | 572 | 258 | 238 | 185 |  |
|  |  |  |  |  |  |  |
| Legend: | >1000 | 999-100 | 99-20 | <20 |  |  |

Heatmap displaying ID_50_ from 51 chronic donors (rows) tested against 5 *env*-pseudoviruses (columns). Neutralization data were expressed as ID_50_ (reciprocal plasma dilution that neutralized 50%) of each psV**.** Plasma titers of <20 were assigned a value of 10 for GMT calculations.
